# Discriminating Different Classes of Toxicants by Transcript Profiling

**DOI:** 10.1289/txg.7036

**Published:** 2004-07-01

**Authors:** Guido Steiner, Laura Suter, Franziska Boess, Rodolfo Gasser, Maria Cristina de Vera, Silvio Albertini, Stefan Ruepp

**Affiliations:** ^1^Non-Clinical Drug Safety and; ^2^Bioinformatics, F. Hoffmann-La Roche Ltd., Basel, Switzerland

**Keywords:** liver, microarray, predictive toxicology, rat, support vector machines, toxicogenomics

## Abstract

Male rats were treated with various model compounds or the appropriate vehicle controls. Most substances were either well-known hepatotoxicants or showed hepatotoxicity during preclinical testing. The aim of the present study was to determine if biological samples from rats treated with various compounds can be classified based on gene expression profiles. In addition to gene expression analysis using microarrays, a complete serum chemistry profile and liver and kidney histopathology were performed. We analyzed hepatic gene expression profiles using a supervised learning method (support vector machines; SVMs) to generate classification rules and combined this with recursive feature elimination to improve classification performance and to identify a compact subset of probe sets with potential use as biomarkers. Two different SVM algorithms were tested, and the models obtained were validated with a compound-based external cross-validation approach. Our predictive models were able to discriminate between hepatotoxic and nonhepatotoxic compounds. Furthermore, they predicted the correct class of hepatotoxicant in most cases. We provide an example showing that a predictive model built on transcript profiles from one rat strain can successfully classify profiles from another rat strain. In addition, we demonstrate that the predictive models identify nonresponders and are able to discriminate between gene changes related to pharmacology and toxicity. This work confirms the hypothesis that compound classification based on gene expression data is feasible.

Microarray technology is a powerful tool allowing simultaneous investigation of gene expression changes of thousands of genes in response to various stimuli. Large-scale and even whole transcriptome analyses have successfully been applied in various fields including variation in budding yeast (Brem et al. 2002), development of *Drosophila melanogaster* (Arbeitman et al. 2002), variation in primates (Enard et al. 2002), and human cancer (Ramaswamy et al. 2003). Class identification and prediction of defined end points using gene expression arrays have shown promising results in oncology (Alizadeh et al. 2001; Ramaswamy et al. 2001; Van de Vijver et al. 2002).

The application of gene expression analysis in toxicology has led to the emergence of the discipline of toxicogenomics. We anticipate that toxicogenomics will greatly improve the sensitivity, accuracy, and speed of toxicologic investigations. Toxicogenomics assumes that toxicity is accompanied by changes in gene expression that are either causally linked or represent a response to toxicity. Indeed, researchers have been able to link toxicity with expression changes of single genes or whole groups of genes (Hamadeh et al. 2002c; Ruepp et al. 2002; Suter et al. 2003).

A transcriptome-wide overview of altered expression patterns can assist the mechanistic understanding of underlying changes induced by chemicals (Hamadeh et al. 2002b). This requires a comprehensive knowledge of the biological system under investigation, and only known genes are considered for analysis. This functional approach is also promising for the generation and testing of toxicity hypotheses (Donald et al. 2002; Zhang et al. 2002) or the identification of perturbed pathways (Wang et al. 1999; Zimmermann et al. 2003). Furthermore, identification of toxic mechanisms is valuable for risk assessment because it allows extrapolation of the hazard in humans.

Predictive toxicology is based on the hypothesis that similar treatments leading to the same end point will share comparable changes in gene expression. Several investigators have used gene expression profiling for the classification of toxicants in rodents (Bulera et al. 2001; Hamadeh et al. 2002a; Thomas et al. 2001; Waring et al. 2001b). These studies varied in design and number of compounds investigated, but all indicated the potential of toxicogenomics in predictive risk assessment.

A major challenge in predicting toxicologic end points based on transcriptional data lies in discriminating changes due to interanimal variation or experimental background noise from treatment-related changes. Compounds may directly affect expression of certain well-characterized, compound-specific genes. These compound-specific genes are not suited for discrimination between different classes of compounds. Drugs, in contrast to other toxic substances, have pharmacologic as well as toxicologic effects that might affect gene expression. These two effects can, but need not, be related. Despite these confounding factors, gene expression analysis after treatment with various compounds that result in the same toxicologic end point should enable identification of a toxic fingerprint.

Various methods are used to analyze large-scale gene expression data. Unsupervised methods widely reported in the literature include agglomerative clustering (Eisen et al. 1998), divisive clustering (Alon et al. 1999), K-means clustering (Everitt 1974), self-organizing maps (Kohonen 1995), and principal component analysis (Joliffe 1986). Support vector machines (SVMs), on the other hand, belong to the class of supervised learning algorithms. Originally introduced by Vapnik and co-workers (Boser et al. 1992; Vapnik 1998), they perform well in different areas of biological analysis (Schölkopf and Smola 2002). Given a set of training examples, SVMs are able to recognize informative patterns in input data and make generalizations on previously unseen samples. Like other supervised methods, SVMs require prior knowledge of the classification problem, which has to be provided in the form of labeled training data. Used in a growing number of applications, SVMs are particularly well suited for the analysis of microarray expression data because of their ability to handle situations where the number of features (genes) is very large compared with the number of training patterns (microarray replicates). Several studies have shown that SVMs typically tend to outperform other classification techniques in this area (Brown et al. 2000; Furey et al. 2000; Yeang et al. 2001). In addition, the method proved effective in discovering informative features such as genes that are especially relevant for the classification and therefore might be critically important for the biological processes under investigation. A significant reduction of the gene number used for classification is also crucial if reliable classifiers are to be obtained from microarray data. A proposed method to discriminate the most relevant gene changes from background biological and experimental variation is gene shaving (Hastie et al. 2000). However, we chose another method, recursive feature elimination (RFE) (Guyon et al. 2002), to create sets of informative genes.

The liver is a primary site for drug metabolism and is frequently involved in adverse drug reactions. Thus, hepatotoxic compounds were chosen for our toxicogenomic studies. In this study 28 hepatotoxic compounds and 3 nonhepatotoxic compounds were investigated. Time-matched controls dosed with the corresponding vehicles were used to allow discrimination between temporal and compound-induced changes. This is essential for large-scale transcriptome analysis, as extensive circadian gene expression patterns have recently been reported in the liver and heart of the mouse (Kita et al. 2002; Panda et al. 2002; Storch et al. 2002).

Depending on the substance and category of toxicity, different time points were chosen for classification, as manifestation of toxicity was observed earlier for certain compounds than for others. Clinical chemistry, hematology, and histopathology were used to assess toxicity of each individual animal.

Models for discrimination of toxic and nontoxic substances as well as models specifying the category of toxicity were built using data from a variety of toxicity studies. The hypothesis that unknown blinded compounds could accurately be classified based solely on gene expression profiles was subsequently tested. In the majority of cases, SVMs were able to predict toxicity as well as the mode of toxicity. The potential for obtaining the same level of predictivity with only a small number of carefully selected genes was investigated. This subset of genes includes potential biomarkers for hepatotoxicity.

## Materials and Methods

### Animal Treatment

Permission for animal studies was obtained from the local regulatory agencies, and all study protocols were in compliance with animal welfare guidelines. Male HanBrl:Wistar rats approximately 12 weeks of age (300 g ± 20%) were obtained from BRL (Füllinsdorf, Switzerland). The animals were housed individually in Macrolone (Tecniplast GmbH, Hohenpeissenberg, Germany) cages with wood shavings as bedding at 20°C and 50% relative humidity in a 12-hr light/dark rhythm with free access to water and Kliba 3433 rodent pellets (Provimi Kliba AG, Kaiseraugst, Switzerland). For the WY14643 study, male Sprague-Dawley Crl:CD(SD)IGS.BR rats approximately 6 weeks of age (200 g ± 20%) were obtained from Charles River Ltd. (Margate, U.K.)

Animals were dosed with test compounds or the corresponding vehicles orally or by ip, iv, or sc injections and sacrificed at specified times by CO_2_ inhalation (Table 1). Immediately preceding sacrifice, terminal blood samples for clinical chemistry investigations were collected from the retroorbital sinus. Liver samples from the left medial lobe were removed immediately and placed into RNALater (Ambion, Austin, TX, USA) for RNA extraction and gene expression analysis (Table 1). The exposure period for each compound was based on reports in the literature and results from pilot studies using histopathology and clinical chemistry anchoring to assess toxicity. Thus, for unknown compounds best results are expected if several time points (e.g., 6 hr, 1 day, and 1 week) are tested.

### Clinical Chemistry

The following determinations were made from the serum: blood urea nitrogen (BUN), alanine aminotransferase (ALT), aspartate aminotransferase (AST), γ-glutamyltransferase (GGT), lactate dehydrogenase (LDH), sorbitol dehydrogenase (SDH), alkaline phosphatase (ALP), 5′-nucleotidase (5′-NT), glutamate dehydrogenase (GLD), urea, glucose, creatinine, bilirubin, total protein, albumin, globulins, total cholesterol, triglycerides, phospholipids, fatty acids, bile acids, sodium, potassium, chloride, calcium, and phosphorus.

### Histology

Representative liver samples were fixed in 10% neutral-buffered formalin. One additional liver sample from the cranial half of the left lateral lobe was placed in Carnoy fixative for glycogen staining. All samples were processed using routine procedures and embedded in Paraplast (Sherwood Medical Ltd., Tullamore, Ireland). Tissue sections approximately 2–3 μ were cut and stained with hematoxylin and eosin or periodic acid-Schiff for glycogen. Fat Red 7B stain (Fluka, Buchs, Switzerland) was performed on frozen formalin-fixed sections to visualize lipid deposits.

### Sample Preparation and Hybridization

RNA isolation, processing, and hybridization were essentially carried out as recommended by Affymetrix (Affymetrix, Santa Clara, CA, USA) with minor modifications [Supplemental data (http://ehp.niehs.nih.gov/txg/members/2004/7036/7036supplement.pdf)].

### Data Acquisition and Preprocessing

Primary data were obtained by laser scanning (Hewlett Packard, Palo Alto, CA, USA) and collated using the Affymetrix Microarray Suite Version 5.0 software (Affymetrix). Before performing any downstream analysis, data were preprocessed in a standardized way. First, the gene expression values of every single microarray experiment were rescaled to a mean value of zero and a standard deviation of 1 to establish comparability across all samples. Because single outlying expression values occur rather frequently and are likely to affect any analysis method, a modified version of the Nalimov outlier test (Kaiser and Gottschalk 1972) was applied to identify these potential artifacts. Expression values reported as outliers were replaced by the respective mean values. The test was performed separately for each classification group (i.e., class of toxicity). In contrast to the published method, our modified version does only one round of outlier removal rather than multiple iterations. A normal distribution model is calculated for the expression levels to be tested, and outliers are removed at a 99% confidence level. As a final preprocessing step, the expression values were rescaled so that the expression of each single gene across multiple arrays has a mean value of zero and a standard deviation of 1. This transformation increases the numerical stability of the SVM algorithm and facilitates the assessment of the relative importance (weight) of single genes within a reduced feature set. Again, this was performed separately for each classification group.

### Support Vector Machines

A detailed introduction into theory and application of SVMs is beyond of the scope of this article. We refer the interested reader to the available literature (Cristianini and Shawe-Taylor 2000; Schölkopf and Smola 2002) and the Supplemental Data(http://ehp.niehs.nih.gov/txg/members/2004/7036/7036supplement.pdf)].

All SVM classifications were based on the free available software package LIBSVM, 2.36, which was downloaded from the World Wide Web (Chang and Lin 2001). The source code was modified according to our needs and compiled to run on the operating system IRIX, version 6.5, (Silicon Graphics, Inc., Mountain View, CA, USA). Extensions such as parameter optimization, feature selection, enhanced cross-validation (CV) options, the one-versus-all training scheme, and report generation were implemented in a C library on top of LIBSVM.

### Choice of Parameters

A linear kernel *k*(**x***_i_,***x***_j_*) *=* 〈**x***_i_,***x***_j_*〉 was chosen for the SVM, as higher order correlation functions could easily lead to overinterpretation of the data, given the unfavorable ratio of features and replicates. LIBSVM offers two different SVM formulations for classification: C-SVM and υ-SVM. These formulations use different parameters for adjusting the accuracy versus margin tradeoff but should produce comparable solutions. We tried both formulations and tuned their respective parameters for optimal CV performance.

To handle the multiple class situation, we applied the one-versus-all training paradigm. Using this approach, a set of binary SVMs is created, each of which separates the samples of one class (positive examples) from all remaining training data (negative examples). Because the number of negative examples usually outweighs the number of positive examples in this scheme, there is always a risk of losing sensitivity for the smaller class. However, practice showed that no additional class bias had to be introduced after appropriate values for the C or υ parameter had been determined for each single SVM. Optimization of these crucial parameters was done in an iterative manner. We typically started with either a C value set to 1.0 or a υ value of 0.5 and performed a complete gene selection run. Optimization of SVMs using different feature numbers suggested improvements in the initial settings as well as a sensible range for the parameters. Feature selection was then repeated with the new settings, and individual SVMs were again tuned to determine good parameters for different gene numbers. This process led to a noticeably improved classification performance.

### Classifier Validation

The predictive power of individual SVMs was primarily rated by their CV performance. However, as our main interest was to estimate the generalization properties of classifiers with respect to new compounds, we did not select the frequently applied leave-one-out or randomization-based schemes. Instead, all microarrays that resulted from the treatment with a certain compound (regardless of dose and time point) were left out as a whole group in one CV cycle. Whenever CV is combined with feature selection, special care must be taken to avoid any bias leading to over-optimistic performance estimates. Therefore, we applied external CV exclusively where feature selection was done separately for each group of left-out examples, thereby avoiding the use of information from the excluded examples in the feature selection process. Although the final classifier is built on all available training examples, the described method was used to determine the optimal number of genes as well as the parameter settings. As a consequence, we expect that the resulting classifiers are less influenced by the given selection of compounds and that CV provides a more realistic estimate for the generalization on new compounds.

Quantitative measures for training and CV performance were sensitivity and specificity values as well as the Matthews correlation coefficient (MCC),


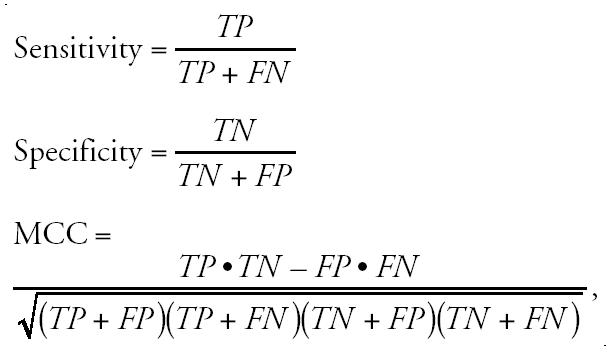


where *TP* = number of true positives, *TN* = number of true negatives, *FP* = number of false positives, and *FN* = percentage of false negatives. The MCC is commonly used as a measure of the predictive power of a system that gives categoric variables as output (Matthews 1975). It was our main performance indicator.

When several SVMs showed exactly the same CV result, performance on the training set was also taken into account. If this still yielded equal results, we finally selected the simplest model (i.e., smallest number of support vectors and smallest number of features).

### Gene Selection

Although SVMs can easily tolerate the high-dimensional gene space typical of microarray studies, most of the features are usually irrelevant for the classification task and only introduce noise. To obtain a meaningful decision function that generalizes well, the number of variables must be reduced as much as possible.

Various methods exist for selecting discriminating features for classification purposes; most deal with variables individually. RFE overcomes some deficiencies of this univariate approach (Guyon et al. 2002). Basically, RFE is a greedy backward elimination method. Starting with all features (except for Affymetrix control genes), a ranking is produced based on the relative importance of a particular feature in the SVM decision function. A certain fraction of the least important variables is then removed, and the process is repeated iteratively until the feature list is empty. The precise order of features might change from iteration to iteration. Because of the multivariate properties of the SVM algorithm, each feature ranking takes into account (at least to some extent) correlations between single variables. Evaluating the classification performance at each step makes it possible not only to identify a suitable subset of descriptors but also to determine how many of them are actually needed for a reliable classification. Redundant features also tend to be eliminated during RFE, typically resulting in very compact feature sets (Guyon et al. 2002).

We implemented RFE on top of the libsvm software. In the beginning, a user-definable fraction of the least important genes is removed in each iteration. After reaching a certain threshold number, only one more gene is eliminated in each step. We experimented with several values for the fraction and lower threshold values to further improve the classification performance of our classifiers.

### Presentation of Support Vector Machine Results

A binary SVM discriminating between two classes is trained by presenting the training samples of one class (A) as positive examples while samples belonging to the other class (B) act as negative examples. An SVM prediction (i.e., the value of the decision function when a new data example is tested) is simply a real number called the discriminant. If the discriminant is positive, the example is considered belonging to class A. Similarly, a negative number would indicate membership in class B. The absolute value of the discriminant can be regarded as a measure of confidence for the classification.

**Figure 1:**
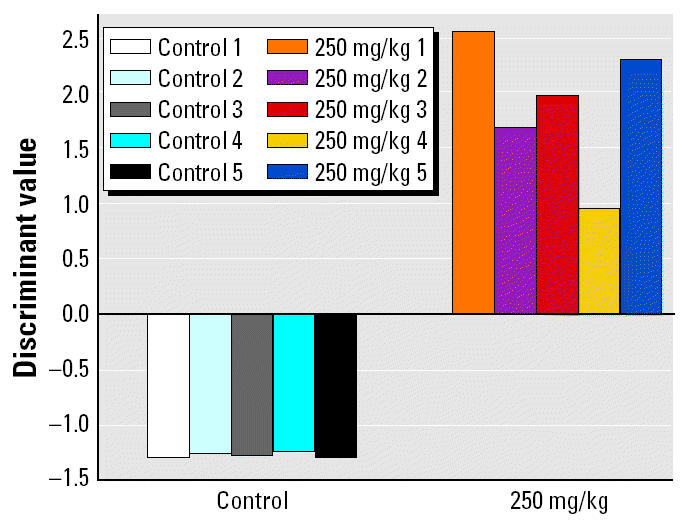
Classification of five vehicle control or five WY14643-treated rats. Gene expression profiles of Sprague-Dawley rat livers treated either with vehicle or WY14643 were assessed with a model built exclusively on data from Wistar rats. Results of the SVM for peroxisomal proliferation are shown. All profiles from treated rats yield clearly positive discriminants, indicating that the transcriptional changes identify the substance to cause peroxisomal proliferation. Controls have clearly negative values, indicating no match with the fingerprint of the peroxisomal proliferation class.

If there are more than two distinct classes, several binary classifiers must be combined to obtain a prediction for a new sample. When applying the one-versus-all scheme (see above), *n* classifiers have to be created for *n* classes. A new data example is then tested with all these SVMs and therefore the result consists of *n* real values from which the most probable class assignment must be inferred. Classification results can be presented as plots of discriminant values that were obtained from a set of SVMs (Figure 1). A unique assignment is possible if only one SVM produces a positive output for a certain sample. If a treatment group is not classified uniformly, we assign the corresponding compound to a category by majority vote, with 60% as the cutoff.

**Figure 2:**
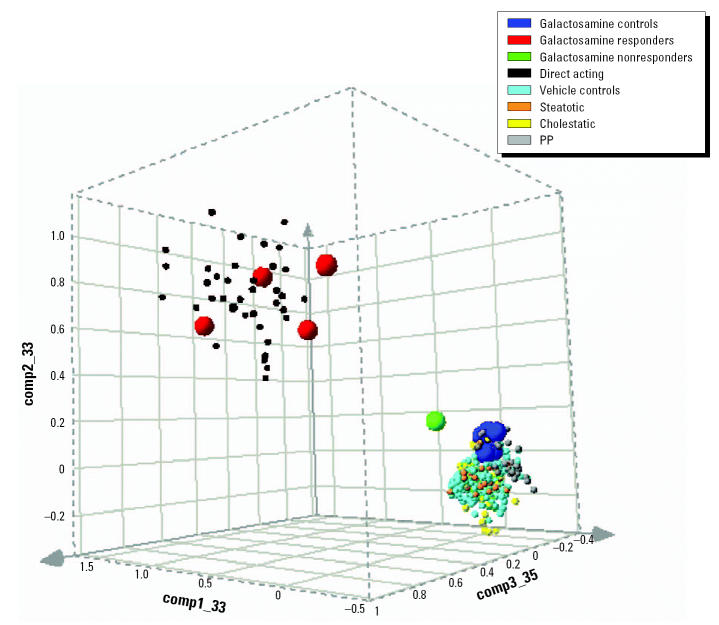
Identification of nonresponding animal. Gene expression profiles from galactosamine-treated rats and vehicle controls were tested using the 4MOT model. Results from the direct-acting SVM (based on 104 genes) are projected onto a three-dimensional coordinate system for better visualization [Supplemental data (http://ehp.niehs.nih.gov/txg/members/2004/7036/7036supplement.pdf)]. Top left: gene expression profiles from direct-acting compounds. Bottom right: profiles from the remaining categories cluster together. Classification results are in line with histopathology and clinical chemistry data. The shift of the nonresponding animal toward the direct-acting group is a hint that gene expression profiling could be more sensitive than classical end points used in this study.

Sometimes two- or three-dimensional scatter plots were produced for visualizing the class separation of one model (Figure 2). These diagrams map all training and test examples into one coordinate system and often reveal some (expected or unexpected) internal structure of the data such as subclusters or single outliers. The dimensionality reduction is achieved by plotting linear combinations of features against each other. Coefficients are obtained from the SVM decision function [Supplemental data (http://ehp.niehs.nih.gov/txg/members/2004/7036/7036supplement.pdf)].

## Results

### Histopathology and Clinical Chemistry—Profiles Used for Training Support Vector Machine Models

We used SVMs as a supervised learning method to generate classification rules. It was of crucial importance to provide training labels on the basis of solid evidence. Therefore, a complete serum chemistry profile and liver histopathology were performed on virtually all rats treated with various model compounds or the appropriate vehicle controls. This information in conjunction with published data provided the basis to allocate gene expression profiles to a specific training class (Table 1).

### Gene Expression Analysis

Gene expression profiles from individual rat livers treated with vehicle or test compounds were analyzed using the Affymetrix U34A GeneChip. All microarrays included in the analysis fulfilled our established quality parameters [Supplemental data (http://ehp.niehs.nih.gov/txg/members/2004/7036/7036supplement.pdf)]. All treatments caused transcriptional changes with respect to their corresponding time-matched controls. In all studies, > 150 genes were expressed above background and showed at least a 2-fold modulation with a *p*-value < 0.05 (two-tailed, unpaired *t* test).

### Assessment of Time Effects in Vehicle Control–Treated Rat Livers (Early versus Late)

Supervised analysis of gene expression data suffers if parameters other than the investigated effects correlate with the classes for which one tries to identify typical finger-prints. The studies evaluated in this article differed in vehicle, application route, and time point (Table 1). We assumed that time-dependent effects could be the confounding factor with the most noticeable impact on the results. Thus, we analyzed gene expression patterns from vehicle-treated animals (i.e., controls) at various time points. A classification attempt was made using the same time points used for the toxicity classifications in this article (early class is 6 hr, late class is 24 hr up to several days). We obtained a prediction accuracy of 70% and an MCC of 0.41, whereas random shuffling of the analyzed microarrays gave MCC values close to zero, indicating that the observed variations can be attributed to time effects. Using this approach, 14 genes were selected as the best set of discriminative features. These results confirmed that there are indeed some observable time-related effects [Supplemental data, Table 1 (http://ehp.niehs.nih.gov/txg/members/2004/7036/7036supplement.pdf)]. However, because time points of the control microarrays typically vary within one class of toxicity, we expected the SVM to identify the time-dependent genes as not relevant for the toxicity predictions. Actually, only one gene of this subset appeared in one toxicity classifier (rc_AA799616_at) but with a low weight.

### Effect of the Rat Strain

Because different rat strains are widely used in toxicology, we investigated the effect of strain differences of Wistar and Sprague-Dawley rats for classification based on transcript profiles. Our database consisting of Wistar rat data was used to generate an SVM. Subsequently, gene expression profiles from vehicle control and WY14643-treated Sprague-Dawley livers were used to assess whether the model would correctly classify individual animals from another rat strain. All five controls were clearly identified as controls (Figure 1). Their transcript profiles yielded negative discriminants for all SVMs except the control SVM, where positive values marked those profiles as controls. Animals treated with 250 mg/kg WY14643 were unanimously assigned to the peroxisomal proliferator class. Here, discriminant values were positive only for the peroxisomal SVM and negative for all other categories. As those results indicated that gene expression profiles from Sprague-Dawley and Wistar rats are comparable, transcript profiles from WY14643 were included in further models.

### Generation of Toxic/Nontoxic and Multitoxicity Models

We generated a binary classifier for the discrimination of vehicle controls and animals treated with a toxic compound. In addition, to predict the mode of action, multitoxicity models were also created. For both the binary and the multiclass case, we used the same data set for training the SVMs, applying either the C-formulation or the υ-formulation of the algorithm. To extract smaller sets of truly discriminative genes, we integrated RFE in the process of model building. This enabled us to study performance parameters such as sensitivity and specificity under CV in relation to the number of genes used.

All the models were evaluated with an external CV scheme that omits a whole treatment group (typically five animals) per cycle. Therefore, a complete RFE run had to be carried out for each of the 60 groups (see “Materials and Methods”). Furthermore, all SVMs were validated with an independent test set that contained different doses and time points of the same substances used for training as well as some new compounds.

The results obtained were similar for C-SVMs and υ-SVMs, although the number of used genes at the point of optimal performance seemed to be smaller for the υ-SVM. However, C-SVM most often outperformed υ-SVM in terms of classification accuracy. Of all 26 toxic substances, C-SVM could not detect 4, whereas υ-SVM missed 6 compounds. A clear majority of the control groups were correctly classified under CV. In this respect there was no pronounced difference between the two formulations.

### Toxic/Nontoxic Model

Results for the binary toxic/nontoxic classification are summarized in Table 2. The test set of 63 vehicle-control groups demonstrates how well those models generalize on previously unseen data [details in Supplemental data (http://ehp.niehs.nih.gov/txg/members/2004/7036/7036supplement.pdf)]. Not every single microarray, but all groups were correctly identified as controls using the described voting procedure. Almost 90% of the toxic test groups were correctly classified as toxic using C-SVM. The model did not produce any false-positive predictions. However, there were some false-negatives, as not all toxic treatments could be recognized as toxic.

### Multiclass Model

As the next step we aimed at predicting the mode of toxicity. For this purpose, a control class and three categories of toxicity were initially defined: cholestasis, steatosis, and direct acting. Subsequently, we added peroxisome proliferator-activated receptor α (PPAR-α) agonists as a separate class without any loss in prediction accuracy (Table 3). We refer to this as the 4-modes-of-toxicity (4MOT) model. This is an imperfect simplification of the classification task, as some of the compounds show more than one form of hepatotoxicity, depending on dose and time. Therefore, time points at which a specific toxicity was most apparent were selected for the analysis.

**Figure 3:**
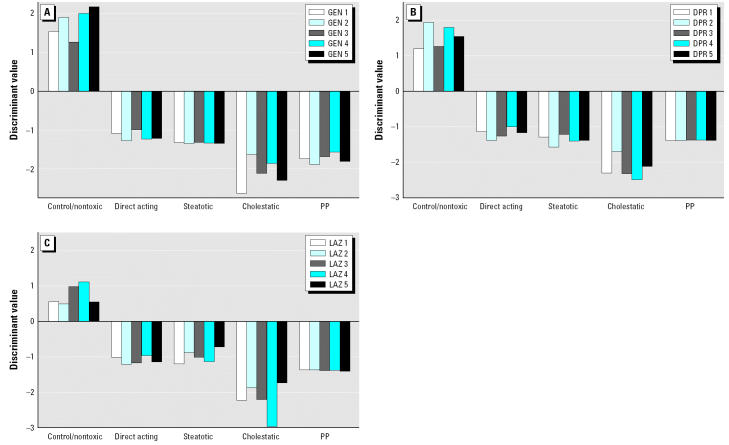
Assessment of gentamicin, deprenyl, and lazabemide. Animals were treated with a high dose of (*A*) gentamicin (GEN; 100 mg/kg sc, 24 hr), (*B*) deprenyl (DPR; 20 mg/kg/day, 4 days), or (*C*) lazabemide (LAZ; 1,000 mg/kg/day, 4 days). No hepatotoxicity was detected with any of the three treatments. However, nephrotoxicity was evident in GEN-treated animals. Gene expression signatures in liver tissue were related to pharmacology without association to hepatotoxicity. Thus, GEN, DPR, and LAZ were correctly identified as nontoxic. Classification of those animals with the controls is indicated by the positive discriminant values for the control SVM.

**Figure 4:**
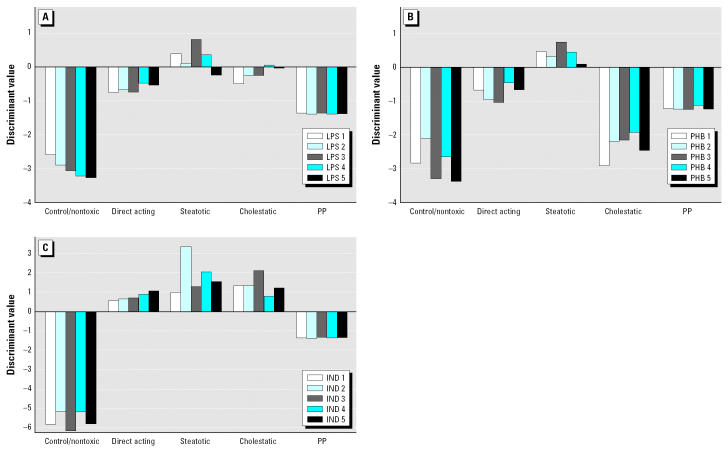
Classification of lipopolysaccharide, phenobarbital, and indomethacin. Abbreviations: IND, indomethacin; LPS, lipopolysaccharide; PHB, phenobarbital. (*A*) Animals were treated with an acute dose of LPS (4 mg/kg iv) and assessed after 6 hr. Four animals were classified as steatotic and one animal as cholestatic (animal 5). (*B*) Rats were dosed with PHB (80 mg/kg po) and assessed 24 hr thereafter. All five animals were considered steatotic. Rats treated with LPS or PHB had very low positive discriminant values for the toxicity categories, indicating no good fit with the representative data in the predictive model. However, the large negative discriminant values of the control SVMs in *A*, *B*, and *C* clearly indicate that all animals were treated with a toxicant. (*C*) Animals were treated with a high dose of IND (5 mg/kg po) and assessed after 1 week. Positive scores were obtained for three different toxic categories. Obviously, the profiles match some characteristics of the finger-prints of all three classes at the same time.

We generated five different SVMs following the one-versus-all approach, that is, each of the models was trained to discriminate between a certain class of toxicity and the set union of all other expression profiles. In a first step, the individual classifiers were built and optimized separately using the same CV procedure described before. Subsequently, a class assignment for each single microarray in the training or test set was done by combining the output of the five models (Tables 4 and 5). In most cases the prediction was unanimous, that is, just one SVM delivered a positive discriminant and the others returned negative values (e.g., Figure 3A–C). In cases where a profile obtained more than one positive discriminant value or only negative numbers, the biggest value determined the classification (e.g., Figure 4). The optimal gene number for classification depends on the category of toxicity. For example, peroxisomal proliferation/PPAR agonists could be recognized with one single marker gene. Nevertheless, the final classifier used four features because of our strategy of simplifying the model by also minimizing the number of support vectors (see “Materials and Methods”). The four top probe sets represent only two distinct genes, acetyl-Coenzyme A acyltransferase 1 (peroxisomal 3-oxoacyl-Coenzyme A thiolase) and cytochrome P450 4A1. Both genes are well-known PPAR-α–responsive genes, and the corresponding upregulation has been described extensively in the literature (Hansmannel et al. 2003; Lee et al. 2003).

The model for the control group required the most features (122). Performance again was rather similar for υ-SVM and C-SVM. In the case of υ-SVM, 274 distinct features were used altogether for discriminating among the five classes of toxicity. However, a reduction to 86 features did not lead to a significant loss in predictivity, indicating that this set could be used for an assay in a 96-well format (data not shown).

Categories of toxicity differ not only in optimal feature number but also in prediction accuracy. Under CV as well as in the test set, all toxicant categories are recognized with a very high specificity, whereas the controls are identified with a high sensitivity. This means that our model produces virtually no false-positive outcomes but at the cost of some false-negative results. All treatment groups within the direct-acting category are either correctly classified or, in the case of aflatoxin, at least recognized as treated with a toxic substance (Table 4). Phalloidin is another example that was identified as toxic, but profiles were classified into two toxicity categories—cholestatic and direct acting. Amiodarone, glibenclamide, and chlorpromazine 1 were not recognized as toxic. Classification of our test set again confirmed the good performance of the model. The classification of 332 test control microarrays with an error rate of 0.6% is remarkable. Using the criteria described above, the success rate in classifying the corresponding 63 control groups is 100% (as seen before in the binary classification).

### Identification of Nonresponding Animals

Galactosamine treatment of rats usually leads to hepatitis associated with necrosis and inflammation. Animals were treated once with 400 mg/kg galactosamine or vehicle only and sacrificed after 24 hr. In four of five galactosamine-treated animals, there was clear evidence of toxicity assessed by hematology, clinical chemistry, and histopathology; one animal was a nonresponder. Gene expression profiles of individual animals were tested using the 4MOT model described previously. Classification results are in perfect agreement with the assessment using conventional end points. However, gene expression profiling seems to be more sensitive than clinical chemistry and histopathology, as the data point corresponding to the nonresponding rat is clearly shifted toward the direct-acting group (Figure 2).

### Pharmacologic Effects Are Differentiated from Toxicologic Effects

Pharmacologically active substances can alter gene expression, but a predictive model for hepatotoxicity should not confuse a substance with a desired pharmacologic effect with an unwanted toxic outcome. Three nonhepatotoxic but pharmacoactive substances were tested with the SVM models. Although 100 mg/kg gentamicin (sc) led to nephrotoxicity at 24 hr, no hepatotoxicity was associated with it, nor was hepatotoxicity detected with deprenyl or lazabemide. All three nonhepatotoxic substances were correctly classified as nontoxic using both, the toxic/nontoxic as well as the 4MOT model (Figure 3). These results show that our toxicity classifiers can distinguish well between pharmacologic effects without toxicity and toxicologically relevant transcriptional changes.

### Classification of Hepatotoxic Compounds with Mechanisms of Toxicity not Represented in Our Database—Lipopolysaccharide, Phenobarbital, and Indomethacin

Compounds with mechanisms of toxicity (MOTs) not represented in our training set were used to investigate how they would be classified by our models. The toxic/nontoxic model had the easier task, as dissimilarity with control profiles would already indicate some toxicity-related abnormality in gene expression. The 4MOT model had to classify an unrepresented profile to one of the five available classes.

Lipopolysaccharide (LPS) (4 mg/kg iv) was investigated 6 and 24 hr after dosing and identified as toxic by the toxic/nontoxic model. The 4MOT model classified four animals as steatotic and one as cholestatic after 6 hr (Figure 4A). After 24 hr four animals were also classified as steatotic and one as direct acting. No sample was misclassified as a control. Phenobarbital (80 mg/kg ip) was also investigated at 6 and 24 hr. At 24 hr all animals fit into the steatotic category (Figure 4B). At 6 hr, four profiles were most similar to the steatotic group and one to the cholestatic group. In this case most discriminant values were very low, indicating differences with respect to the existing classes. Another interesting example was indomethacin, which was administered either as a single high dose (20 mg/kg po; sample collection at 6 or 24 hr) or as a repeated low dose (5 mg/kg po; daily dosing during 1 week). In the liver, minimal to slight hepatocellular hypertrophy and decreased glycogen deposition were observed in animals treated with 20 mg/kg at 24 hr and in animals treated for 1 week with 5 mg/kg/day. The repeated dosing also caused tubular dilation in the kidney and erosive and/or ulcerative inflammations in the gastrointestinal tract. At 6 hr the substance was classified as predominately cholestatic and at 24 hr clearly as steatotic. After 7 days of dosing, three animals were classified as steatotic and two as cholestatic. These profiles had positive discriminants for three toxicity categories (cholestatic, steatotic, direct acting). This indicates that indomethacin is different from our predefined toxicity categories and displays mixed toxicity (Figure 4C). Most important, a very clear dissimilarity from the control group indicated that the indomethacin-treated animals had been exposed to a toxic compound, although the mode of toxicity could not be unequivocally defined.

## Discussion

### Gene Expression Profiling

The present work aimed to provide evidence that transcript profiles can be used to distinguish compound-treated rat livers from controls and to discriminate between different MOTs. Rats were treated with a variety of vehicles, and hepatotoxic or non-hepatotoxic but pharmacologically active compounds. We focused on hepatotoxicity, as the liver is a main target for toxic reactions. Various questions were addressed in the context of predictive toxicity modeling, including sanimal variability, rat strain differences, effect of time, and discrimination of pharmacologically from toxicologically induced gene changes.

Several authors have described the use of gene expression profiling to classify toxicants in rodent liver and thereby demonstrated the potential of toxicogenomics in predictive risk assessment (Bulera et al. 2001; Hamadeh et al. 2002a; Thomas et al. 2001; Waring et al. 2001b). We used a larger number of compounds and selected a different bioinformatics approach to analyze the data. New in this study is the modeling of different categories of toxicity in conjunction with numeric measures for the classification confidence. Our results demonstrate that for different compounds with similar MOT, the likely toxicologic end point can be inferred from gene expression profiles using a database of model compounds as a training set. Moreover, we found good correlation of gene expression changes with histopathologic findings. These results are consistent with those of a previously reported study where methapyrilene toxicity correlated with the severity of pathologic changes (Hamadeh et al. 2002c).

### Feature Selection

SVMs can handle very high-dimensional feature spaces, so there is no pressing need to filter out a small number of genes in a first step. In contrast to many published microarray studies, we did not apply strict cutoffs like 2-fold changes, *p*-value thresholds, or similar criteria.. These approaches could easily spoil one of the main advantages of a multivariate classification method such as SVMs, as prefiltering of features by common univariate methods (such as the *t* test) might remove genes that do not reach significance when tested individually but provide useful information when taken together with other, correlated variables. In contrast, RFE allowed us to combine feature selection and model building in a consistent framework, making use of the mutual information between genes (Guyon et al. 2002). We leave it to the method to eliminate noisy, irrelevant variables in the process of forming smaller and smaller subsets of genes with discriminatory power. The approach also helped to avoid the introduction of a feature selection bias, which occurs if information from all experiments is used to reduce the number of genes before any CV is done. However, it is important to remember that the gene lists we obtained are in no way a complete picture of the cellular response but a redundancy-reduced selection of markers that together allow a maximum predictivity.

The relationship between gene number and classification performance was studied using RFE, and subsequently the optimal iteration was chosen. Our results indicate that accurate prediction of toxicity (including the category of toxicity) can be achieved using a small set consisting of a few up to some dozens of features (Table 3). In the case of the 4MOT model, the feature number can be reduced from 274 to 86 without major performance impairment. The observation that more genes do not necessarily translate into higher predictive accuracy is consistent with previous findings (Ramaswamy et al. 2003; Thomas et al. 2001), indicating that it is not necessary to measure the whole transcriptome or thousands of genes to predict toxicity. Once initial experiments have led to an optimized set of relevant informative features, a potentially faster and cheaper assay could be developed providing essentially the same classification performance. Interestingly, using only the selected features for hierarchical clustering also resulted in a toxicologically meaningful result, whereas unsupervised clustering with all genes often failed at classifying the animals according to the criteria of interest (data not shown). However, it is worth mentioning that none of the genes in the final set is guaranteed to act as a good toxicity marker on its own because we do not rank features according to their suitability as single markers (univariate approach) but rather optimize whole subsets of features (multivariate approach). In this setting it is possible that a gene that does not appear differentially expressed in two groups can still contribute useful information by combination with other genes. Therefore, it is often the signature taken as a whole that provides the decisive discriminatory power. Marker gene sets identified with the described method are especially prone to show this effect because of the multivariate nature of SVMs and the tendency of the RFE algorithm to eliminate redundant features from the set (Guyon et al. 2002).

As gene expression analysis can also be applied *in vitro* (Burczynski et al. 2000; Waring et al. 2001a), the question arises whether the list of features obtained could be used in a cell-based assay. This seems questionable, as significant differences in gene expression *in vitro* compared with *in vivo* were reported (Boess et al. 2003). Therefore, we expect that results concerning discriminative features and their weights cannot be directly transferred to *in vitro* classification systems. In addition, the evaluation of the compound effects *in vivo* is especially important when multiple cell types and possibly multiple organs are involved in the toxicologic response.

### Confounding Effects

A crucial issue when using supervised classification methods is that there must be solid evidence for the initial assignment of gene expression profiles to each category. Therefore, we included only microarrays from animals where independent evidence justified allocation to a specific class. In most cases, histopathologic anchoring was used, but clinical chemistry and occasionally additional biochemical assays (triglyceride assays, data not shown) were also considered. Anchoring to conventional end points was the reason for the heterogeneity of time points used in the training procedure. This kind of heterogeneity might act as a confounding factor, introducing signatures not related to the toxicity classification problem itself. Special care must be taken to ensure that these confounding factors do not exhibit decisive influence on the model. The potentially confounding effect of time was addressed first, as several authors have highlighted extensive circadian gene expression changes (Kita et al. 2002; Panda et al. 2002; Storch et al. 2002). For this purpose, the same time points (6 hr, 24 hr, and several days) used within our toxicity models were used to train a two-class SVM model for classification of early or late time points. A classifier based on 14 genes was obtained, but predictivity was far from perfect and resulted in a relatively low MCC of 0.41. (Test MCC values for the toxicity classifiers were all > 0.80.) Although these results confirm some time dependency in our experiments, we have no reason to assume that this strongly affects our toxicity models, as we always combined control profiles from all time points in the same group for training. Together with the fact that none of the genes from the time classifiers appeared at a prominent position (with significant weight) in the toxicity models, these results suggest that there is no distinct time bias. In fact, classification of vehicle controls from the test set (originating from independent studies and including various time points) was correct in more than 99% of the cases, which confirms the absence of time bias for the control component of the classifier.

Wistar, Sprague-Dawley, and Fischer rats are all frequently used in risk assessment. There is ample evidence that those strains vary in their susceptibilities to various toxicants or mutagens (Asamoto et al. 1989; Kulkarni et al. 1996). Therefore, we investigated whether a model built with Wistar rat expression profiles would be predictive for treatment effects in Sprague-Dawley rats. PPAR agonists were chosen for this comparison for pragmatic reasons. At the time we studied proprietary PPAR agonists, we were also involved in the Consortium for Metabonomic Toxicology (COMET), where liver tissue collection of WY14643-treated Sprague-Dawley rats could be included. [COMET has been formed by Imperial College (London) and six major pharmaceutical companies. The objective is to apply metabonomics to the toxicologic assessment of compounds (Lindon et al. 2003).] Treated rats as well as controls fit perfectly into the anticipated classes. The classification was successful despite the additional confounding factor introduced by the fact that the Sprague-Dawley rats were approximately 6 weeks younger than the Wistar rats. This successful class prediction was the rationale for including those expression profiles in our predictive models. As the results suggest, the discriminative transcriptional changes are largely conserved across strains, although the doses required to produce comparable toxicity may vary.

Another confounding factor for the classification task is that pharmaceuticals not only show a toxic effect on gene regulation but might also influence gene expression according to their pharmacologic action. A crucial test for the classification of toxicants based on gene expression profiles is certainly the ability to separate pharmacologic from true toxic effects. Our models succeeded at classifying three pharmacologically active, nonhepatotoxic compounds. In the case of gentamicin, not even the observed nephrotoxicity led to a false prediction of hepatotoxicity. The classification of these three compounds as nonhepato-toxic was not due to a general lack of effects on hepatic gene expression; more than 100 genes were differentially expressed for these compounds, as assessed by fold change together with *t* test (at least 2-fold change and *p*-value < 0.05).

### Mixed Toxicities

All transcript profiles were assigned to a specific category, implying that they fit exactly into one class. However, in reality, substances often cause mixed toxicities. We aimed to allocate substances to the best-fit-ting class, knowing the limitations due to the potential overlap of effects. Our results indicate that characteristic gene expression changes are indeed associated with distinct classes of toxicants. However, as compounds cannot be put into exclusive bins in a strict sense, some substances (aflatoxin, indomethacin, and phalloidin) were predicted to be associated with multiple toxicities. Aflatoxin, for example, needs metabolic activation to exert its toxic effect. It causes generation of reactive oxygen species, lipid peroxidation, glutathione depletion, and necrosis and therefore has a direct effect on cells (Liu et al. 1999). On the other hand, it is a well-known carcinogen (Smela et al. 2001) and is reported to induce both cholestasis (Unger et al. 1977) and steatosis (Amaya-Farfan 1999). Based on classical end points, we decided to allocate aflatoxin to the direct-acting group. The SVM classification of gene expression profiles, however, indicated a greater similarity to cholestatic than to direct-acting compounds. One possible way to address this problem might be to generate several one-versus-control categories and include the aflatoxin samples in both the direct-acting and the cholestatic classes. Another option would be to exclude all compounds from training that do not unambiguously fit into one single category. Reported effects of indomethacin in rats are immediately direct, like adenosine triphosphate depletion in hepatocytes (Masubuchi et al. 1998) and a marked decrease in the hepatic monooxygenase system (Fracasso et al. 1990). Gene expression profiles of rats dosed with indomethacin were classified as cholestatic and steatotic but also matched the direct-acting group. Clinical chemistry supported this mixed toxicity prediction to some extent, as ALP, GGT, AST, and LDH were increased. Histopathology revealed hypertrophy and minimal to slight necrosis, but changes were considered to be adaptive rather than reflecting an adverse effect. In patients, however, cases of cholestasis and steatosis have been reported (Farrel 1994). It remains to be confirmed whether the genomics approach is more sensitive than histopathology in detecting liabilities.

Results classifying galactosamine-treated rats using the multitoxicity model support this hypothesis. Galactosamine treatment leads to hepatitis associated with necrosis and inflammation, but a high degree of interanimal variation is well known (Vomel and Platt 1986). In our study four of five expression profiles were identified as toxic while the fifth was classified as control. This classification as nonresponder was in agreement with absence of findings using conventional end points. However, a three-dimensional plot of the SVM results revealed a shift of the expression profile of the nonresponder toward the direct-acting group (Figure 2 ), suggesting increased sensitivity of the toxicogenomics approach.

### Model Assessment

We used a compound-based external CV scheme (see “Materials and Methods”) to obtain more realistic estimates for the classification performance and to select a model from which we can expect a good generalization power. It has to be kept in mind that the compound database is still limited in size, and we do not know whether our set of substances is a representative sampling of the complete toxicology space. Therefore, we cannot completely rule out some sampling bias, which would render our performance estimates too optimistic. Conversely, our CV procedure intrinsically tends to deliver a rather conservative assessment of the performance, as at least some of the compounds provide vital information that is lost as soon as a whole treatment group is withheld from training. For example, glibenclamide (dosed at 25 mg/kg) was not recognized as a cholestatic compound under CV; the final SVMs correctly classified two of five animals in the test set, despite the fact that these had been treated with a lower dose (2.5 mg/kg) and histopathology was only evident in animals that received the high dose.

Because of the partial overlap of compounds in the training and test set, one would expect a smaller fraction of misclassifications under test conditions than with the more rigorous CV method. This was indeed observed in most cases (Tables 2 and 3) and emphasizes the extent to which interpretation of results depends on the details of the applied evaluation method. Although in this case the number of CV errors can provide information about the generalization ability of a model, the test performance should be regarded as a measure for its consistency with respect to a certain selection of compounds. For our application we clearly wanted to optimize the former; therefore, we used the described CV scheme to select the best SVMs. When we tentatively switched to a more standard, leave-one-out procedure, not a single CV error occurred. However, the test accuracy was significantly decreased, indicating that a classifier with less generalization ability had been generated by this standard CV method.

The current model was based on histopathologic and clinical chemistry data and performs best on data comparable to the training data. If there is no evidence of toxicity (see deprenyl, lazabemide, gentamicin), the gene expression profiles are not wrongly assigned to a toxicity category. Classification of lower but still somewhat toxic doses was successful with dichlorobenzene (1,500 mmol/kg), amineptine (0.25 mmol/kg), acetaminophen (1,000 mg/kg), bromobenzene (1 mmol/kg), Rx50 (1 mg/kg/day), Rx51 (0.13 mg/kg/day), and Rx60 (0.38 mg/ kg/day). Nevertheless, detection of toxic substances applied at subtoxic doses can be successful; examples include Rx10 (125 mg/kg/day) and some animals treated with glibenclamide 2.5 mg/kg. However, it is important to remember that the current model was based on solid pathology and therefore optimized for specificity. If borderline or doses just below detectable pathology were used to generate the model, correct classification at subtoxic doses could be expected in more cases. However, an increase in sensitivity is expected to be paid for by a reduced specificity (i.e., greater number of false positives).

If there were evidence for toxicity, although with a lower histopathologic score and less-pronounced clinical chemistry changes, the model generally performed well, as indicated by the relatively high test MCC values. Examples for successful classification of earlier time points are Rx99 (24 hr) and methylene dianiline (3 hr). However, very early times can often affect a different set of genes than those noted at later times (Heijne et al. 2004; Ruepp et al. 2002).

Although the test set contained some of the same compounds as the training set, the experiments in the test set used lower doses or samples collected at earlier time points. Therefore, the high classification accuracy observed with the test data indicates good sensitivity of our models.

An interesting observation was that the two experiments using chlorpromazine were not equally well classified. Chlorpromazine was expected to have a cholestatic effect at the tested doses (Knodell 1975), but the animals were classified predominantly as nontoxic in the first experiment and as cholestatic in the second experiment. However, these differences in gene expression profiles are in agreement with differences in conventional end points, probably because of biological and/or experimental differences, as both experiments were performed at different sites and with slightly different sample processing protocols.

Summarizing, we demonstrated that classification problems in toxicogenomics can be effectively addressed by a supervised learning approach. We applied SVMs on microarray data from a set of model hepatotoxicants. Combining SVM parameter optimization with a compound-based external CV scheme and (RFE), we were able to obtain accurate classification (i.e., high sensitivity and specificity) of the compounds included in the training set as well as for previously unseen compounds. In addition, RFE allowed us to select a relatively compact subset of probe sets with potential use as biomarkers. Thus, our results show that toxicogenomics is a very powerful tool for classification of compounds according to their toxicity mechanism when a well-designed database is combined with appropriate bioinformatics tools.

Despite these promising results, further investigations must be performed to increase the usefulness of transcript profiling in toxicology. A larger database and refined analysis methods are anticipated to further improve prediction accuracy.

We focused mainly on high doses that led to clear toxicity as assessed by conventional end points. However, it has been reported that a compound affects different genes and pathways depending on the administered dose (Andrew et al. 2003). Thus, a next step will be to include expression profiles from lower doses in the model-building process. Earlier time points should also be considered. This will allow us to assess whether gene expression changes are already indicative of toxic liabilities when standard parameters do not yet detect toxicity. In addition, for classification purposes it is irrelevant whether the gene expression changes considered good discriminants for a toxic response are causally linked to the toxicity. Nevertheless, to gain further insight into a specific MOT, it is valuable to interpret results in a biological context, analyzing the altered pathways and their relationship to observed pathology or phenotype. These investigations could help separate transcriptional changes that are relevant for the mode of toxicity from mere bystander effects.

## Supplementary Material

Supplemental Tables

## Figures and Tables

**Table 1:** Histopathology and clinical chemistry results of rats used included in the SVM training set.

Substance/dose/CAS no./supplier	Vehicle/route of administration	Expected binary class/4-MOT class	Liver histopathology	Serum clinical chemistry
Aflatoxin B_1_	Saline + 0.5%	Toxic/direct	Hepatocellular hypertrophy, apoptosis, inflammation, glycogen depletion, bile duct proliferation	Increased bile acids, bilirubin, AST, ALT, LDH, ALP, 5′-NT
4 mg/kg, 24 hr	DMSO/ip			
1162-65-8				
Sigma				
Bromobenzene	Corn oil/ip	Toxic/direct	Centrilobular to midzonal hepatocellular hydropic swelling, necrosis with mixed inflammation	Increased bilirubin, 5′-NT, albumin; decreased triglycerides
3 mmol/kg, 24 hr				
108-86-1				
Aldrich				
Carbon tetrachloride (CCl_4_)	Corn oil/po	Toxic/direct	Hepatocellular degeneration, single-cell necrosis, inflammation, microvesicular steatosis	Increased GGT, liver triglycerides; decreased glucose, albumin
2 mg/kg, 24 hr				
56-23-5				
Fluka				
Hydrazine	Saline/ip	Toxic/direct	Hepatocellular necrosis with inflammation, mild microvesicular steatosis	Increased 5′-NT
60 mg/kg, 24 hr				
302-01-2				
Sigma				
Thioacetamide	Saline/ip	Toxic/direct	Hepatocellular vacuolation and necrosis	Increased GGT, AST, ALT, ALP,5′-NT; decreased glucose, triglycerides, cholesterol, protein
50 mg/kg, 24 hr				
62-55-5				
Sigma-Aldrich				
1,2-Dichlorobenzene	Corn oil/ip	Toxic/direct	Centrilobular to midzonal hepatocellular hydropic swelling, necrosis with mixed inflammation	Increased ALP, albumin; decreased triglycerides
4,500 mmol/kg, 24 hr				
95-50-1				
Fluka				
Coumarin	Corn oil/po	Toxic/direct	Hepatocellular hypertrophy, single-cell necrosis, lymphocytic infiltration	Increased total protein, GLD
200 mg/kg, 24 hr				
91-64-5,				
Sigma				
Acetaminophen	Saline + 0.5%	Toxic/direct	Centrilobular hepatocellular vacuolation, single-cell necrosis, inflammation	Increased albumin; decreased triglycerides
2 g/kg, 24 hr	DMSO/po			
103-90-2				
Fluka				
Amineptine	Saline/ip	Toxic/steatosis	Hepatocellular microvesicular steatosis, glycogen depletion	Increased GGT, ALP, cholesterol; decreased triglycerides
0.5 mmol/kg/day, 2 days				
57574-09-1				
Servier Laboratories				
Amiodarone	7.5% gelatine/ip	Toxic/steatosis	Hepatocellular microvesicular steatosis, glycogen depletion	Increased GGT, 5′-NT; decreased serum and increased liver triglycerides
100 mg/kg/day, 4 days				
1951-25-3				
Sigma				
Rx74 (Antidiabetic)	Klucel/po	Toxic/steatosis	ND*^a^*	ND*^a^*
250 mg/kg/day, 5 days				
Not available				
Roche				
Rx75 (Antidiabetic)	Klucel/po	Toxic/steatosis	ND*^a^*	ND*^a^*
100 mg/kg/day, 5 days				
Not available				
Roche				
Rx10 (Antidiabetic)	Klucel/po	Toxic/steatosis	ND*^b^*	ND*^b^*
500 mg/kg/day, 5 days				
Not available				
Roche				
Rx99 (5-HT_6_ antagonist)	H_2_O/po	Toxic/steatosis	Hepatocellular microvesicular steatosis	Increased ALT, GGT; increased liver lipids and phospholipids
400 mg/kg/day, 14 days				
Not available				
Roche				
Chlorpromazine 1	Saline/iv	Toxic/cholestasis	ND	Increased bilirubin, glucose; decreased triglycerides
15 mg/kg, 6 hr				
69-09-0				
Sigma				
Chlorpromazine 2	Saline/iv	Toxic/cholestasis	Hepatocellular microvesicular steatosis, glycogen depletion	Increased glucose; decreased triglycerides, protein
15 mg/kg, 6 hr				
69-09-0				
Sigma				
Cyclosporin A	10% intralipid/iv	Toxic/cholestasis	NSF	Increased bile acids, bilirubin, GGT
30 mg/kg, 6 hr				
59865-13-3				
Alexis				
Glibenclamide	7.5% gelatine/iv	Toxic/cholestasis	Hepatocellular hypertrophy	Increased ALT; decreased glucose
25 mg/kg, 6 hr				
10238-21-8				
Roche				
Phalloidin	Saline/iv	Toxic/cholestasis	Hepatocellular necrosis, hemorrhage, glycogen depletion	Increased bilirubin, bile acids, 5′-NT, ALP, AST, ALT, LDH, SDH; decreased cholesterol, phospholipids
0.8 mg/kg, 6 hr				
17466-45-4				
Sigma				
Methylene dianiline	Corn oil/po	Toxic/cholestasis	Single-cell necrosis of bile duct epithelium, inflammation	Increased bilirubin, bile acids, GGT, 5′-NT, glucose, phospholipids
100 mg/kg, 6 hr				
101-77-9				
Fluka				
WY14643	Corn oil/po	Toxic/PP	Increased hepatocellular mitoses, slight glycogen depletion, increased liver weight (7 days)	Increased ALP, glucose, SDH
250 mg/kg, 14 days				
50892-23-4				
Sigma-Aldrich				
				
Rx90 (PPAR-δ agonist)	PBS/po	Toxic/PP	Liver enlargement, diffuse hepatocellular hypertrophy	Increased AST, ALT
180 mg/kg/day, 14 days				
Not available				
Roche				
Rx53 (PPAR-α,γ co-agonist)	PBS/po	Toxic/PP	Increased liver weight, hepatocellular hypertrophy and cytoplasmic granulation	Decreased cholesterol, protein
0.9 mg/kg/day, 14 days				
Not available				
Roche				
Rx60 (PPAR-α,γ co-agonist)	PBS/po	Toxic/PP	Increased liver weight, hepatocellular hypertrophy and cytoplasmic granulation, increased mitoses, single-cell necrosis with mixed inflammation	Increased serum ALP; decreased protein, bilirubin
1.5 mg/kg/day, 14 days				
Not available				
Roche				
Rx51 (PPAR-α,γ co-agonist)	PBS/po	Toxic/PP	Increased liver weight, hepatocellular hypertrophy and cytoplasmic granulation	Increased ALP; decreased cholesterol, bilirubin, protein
0.5 mg/kg/day, 14 days				
Not available				
Roche				
Rx50 (PPAR-α,γ co-agonist)	PBS/po	Toxic/PP	Increased liver weight, hepatocellular hypertrophy and cytoplasmic granulation	Increased ALP, glucose; decreased protein, bilirubin, cholesterol
4 mg/kg/day, 14 days				
Not available				
Roche				

Abbreviations: DMSO, dimethylsulfoxide; ND, not done; NSF, no significant findings; PBS, phosphate-buffered saline; PP, peroxisome proliferator.

a No clinical chemistry or histopathology data were available from animals used for gene profiling, but repeated dosing with this compound in animals used for other measurements resulted in microvesicular steatosis.

b No clinical chemistry or histopathology data were available from animals used for gene profiling. Microvesicular steatosis was not detected in rats with this treatment schedule. However, *in vitro* treatment of primary rat hepatocytes inhibited β-oxidation and resulted in fat accumulation.

**Table 2:** Performance of the toxic/nontoxic models and summarized results of the binary (toxic/nontoxic) classification.*^a^*

Arrays/groups for classification	ν-SVM	C-SVM
Classification under external CV		
26 treatment groups	20 of 26 treatments correct	22 of 26 treatments correct
116 arrays	89 of 116 arrays correct	90 of 116 arrays correct
34 control groups	32 of 34 groups correct	32 of 34 groups correct
163 arrays	154 of 163 arrays correct	154 of 163 arrays correct
Classification of test set		
19 treatment groups	16 of 19 treatments correct	17 of 19 treatments correct
91 arrays	74 of 91 arrays correct	74 of 91 arrays correct
63 control groups	63 of 63 (all groups correct)	63 of 63 (all groups correct)
332 arrays	322 of 332 arrays correct	327 of 332 arrays correct

a During RFE, the least informative 5% of genes were removed in each iteration starting with all features (genes) down to 64 genes. After that, only a single gene was removed in one step. The number of features finally selected was 63 for the ν-SVM and 228 for the C-SVM. In the case of ν-SVM, RFE was carried out with ν = 0.1. The optimized ν of the selected (using 63 genes) is 0.203. For C-SVM we set C to 0.008 during RFE and ended up with C = 0.00429 for the selected iteration. Both SVMs were equally successful in classifying vehicle controls, but the C-SVM was slightly better in identifying toxic treatments.

**Table 3:** Performance assessment of the five SVMs that form the 4MOT model.*^a^*

Class	Features	CV specificity	CV sensitivity	CV MCC	Optimized	Test specificity	Test sensitivity	Test MCC
Classification with υ-SVM								
Direct	101	1	0.86	0.92	0.0377	1	0.75	0.83
PP	4	1	1	1	0.01	1	1	1
Cholestasis	19	0.99	0.6	0.71	0.0193	0.99	0.83	0.82
Steatosis	28	1	0.54	0.72	0.0744	1	0.91	0.95
Control	122	0.78	0.94	0.75	0.111	0.84	0.98	0.86
Classification with C-SVM								
Direct	38	1	0.84	0.9	0.0176	1	0.75	0.83
PP	16	1	1	1	0.0222	1	1	1
Cholestasis	32	0.98	0.57	0.61	0.1	0.98	0.83	0.81
Steatosis	50	0.99	0.67	0.75	0.00869	1	0.91	0.95
Control	228	0.78	0.94	0.74	0.00429	0.8	0.98	0.83

a Results are shown for υ-SVM and C-SVM. The RFE procedure was identical to that described in Table 2. The number of features selected was typically smaller for υ-SVM than for C-SVM. Both types of SVM were comparably successful in classification.

**Table 4:** Classification of individual microarrays and treatment groups in training set and overview of CV and test results for a υ-SVM–based model discriminating between different MOTs.*^a^*

Treatment	Expected toxicity category	CV accuracy (binary)	CV accuracy (4MOT)	Misclassification in 4MOT
Chlorpromazine 1	Cholestatic	1/5*^b^*	1/5*^b^*	4 controls*^b^*
Chlorpromazine 2	Cholestatic	4/5	4/5	1 control
Cyclosporin A	Cholestatic	4/5	4/5	1 control
Glibenclamide	Cholestatic	0/5*^b^*	0/5*^b^*	5 controls*^b^*
Methylene dianiline	Cholestatic	5/5	5/5	–
Phalloidin	Cholestatic	3/5	*2/5*	1 direct acting, 2 controls
Aflatoxin B_1_	Direct acting	2/3	*1/3*	1 cholestatic, 1 control
1,2-Dichlorobenzene	Direct acting	5/5	5/5	–
APAP	Direct acting	3/5	3/5	2 controls
Bromobenzene	Direct acting	5/5	5/5	–
CCl_4_	Direct acting	5/5	5/5	–
Coumarin	Direct acting	5/5	5/5	–
Hydrazine	Direct acting	5/5	5/5	–
Thioacetamide 1	Direct acting	3/5	3/5	2 controls
Rx50 (PPAR-α, γ)	PP	5/5	5/5	–
Rx53 (PPAR-α, γ)	PP	2/4*^b^*	2/4*^b^*	2 controls*^b^*
Rx51 (PPAR-α, γ)	PP	5/5	5/5	–
Rx60 (PPAR-α, γ)	PP	5/5	5/5	–
WY14643	PP	5/5	5/5	–
Rx90 (PPAR-δ)	PP	5/5	5/5	–
Rx99 (5HT_6_)	Steatotic	3/5	3/5	2 controls
Amineptine	Steatotic	4/5	4/5	1 control
Amiodarone	Steatotic	0/5*^b^*	0/5*^b^*	5 controls*^b^*
Rx74 (anitdiabetic)	Steatotic	3/3	3/3	–
Rx75 (anitdiabetic)	Steatotic	2/3	2/3	1 control
Rx10 (anitdiabetic)	Steatotic	3/3	3/3	–

a Predictions for individual microarrays and treatment groups as a whole were obtained using different voting schemes described in the text. A compound-based external CV method was used for the assessment of model quality. The upper part of the table reports the number of microarrays correctly classified under CV conditions, either with correct mechanism of action predicted (column 4) or with at least a toxic effect recognized (column 3).

b Misclassifications.

**Table 5:** Performance summary of the υ-SVM–based model discriminating between different MOTs.

Arrays/groups for classification	Summary
26 treatment groups	20 of 26 treatment groups correct MOT identified
	22 of 26 treatment groups correctly identified as toxic
116 microarrays	85 of 116 microarrays correctly classified
34 control groups	33 of 34 groups correctly identified as vehicle controls
163 microarrays	160 of 163 microarrays correctly classified
Classification of independent test set	
19 treatment groups	15 of 19 treatment groups correct MOT identified
	15 of 19 treatment groups correctly identified as toxic
91 microarrays	74 of 91 microarrays correctly classified
63 treatment groups	63 of 63 (all groups correctly identified)
332 microarrays	330 of 332 microarrays correctly classified
