# Supplementary material for: Discriminating Different Classes of Toxicants by Transcript Profiling
Source: Environ Health Perspect. 2004 Jul 1;112(12):1236–48. doi: 10.1289/txg.7036 (PMC1277117; doi:10.1289/txg.7036)
Supplement: Supplemental Tables [file ehp0112-001236s1.pdf]

SUPPLEMENTAL DATA

SVM algorithm. The fundamental binary classification problem can be regarded as a set of n training examples $\boldsymbol{x}_i \in \Re^m$ $(i=1...n)$ together with their respective class labels $y_i \in \{+1,-1\}$. Each example comprises *m* observable variables (features). The class label indicates whether the corresponding data point is a positive or negative example for a particular subgroup of the data, thus reflecting the prior knowledge we have for the training set. (In our case, one data point x corresponds to an individual microarray with 8799 gene expression values.) Solving the classification problem is equivalent to finding a function $F: \boldsymbol{x} \rightarrow \{+1,-1\}$ whose internal parameters are adjusted in such a way that it can reproduce the correct class labels y_i for each training example $\boldsymbol{x}_i$. In addition, we demand that the solution $F(\boldsymbol{x})$ should not only recognize *the training examples*, but also be able to *generalize*, at least to some extent, on previously unseen data points for which we will use the term *test examples*. $F(\boldsymbol{x})$ is often called a decision function.

Support Vector Machines (SVMs) are one implementation of the concept of a supervised learning algorithm that uses known information about classes and solves the classification problem while aiming to minimize the probability of false classifications for initially unknown test data. The basic ideas of the SVM method and detailed explanations are described in the literature [Cristianini and Shawe-Taylor 2000; Schölkopf et al. 2002].

In its simplest form, the SVM algorithm works by determining a hyperplane

$$f(\boldsymbol{x}) = \langle \boldsymbol{w},\boldsymbol{x} \rangle + b \qquad (1)$$

that separates positive from negative examples directly in the input space of the given data. For the linearly separable problem, it can be shown that is always possible to find an *optimal separating hyperplane* in the sense that the margin (i.e. the distance between the plane and the closest lying data points) is maximized. Just as intuition might suggest, from this approach we also get a solution that minimizes the expectation for classification errors when applying the

classifier on an independent test set. Furthermore it can be proven that the normal vector of the optimal separating hyperplane can always be expressed in the basis of the given training patterns, i.e.

$$\boldsymbol{w} = \sum_i (\alpha_i \, y_i \, \boldsymbol{x}_i) \qquad (2).$$

It is a remarkable observation that in almost all realistic data sets a large fraction, if not most of the coefficients α_i reach a value of zero in the process of optimization. This means that the solution only depends on a relatively small subset of data points which are called *support vectors*. They correspond to the training examples lying closest to the separating hyperplane ("borderline cases"). This *sparseness of the solution* not only gives rise to computational benefits, but also has some important learning theoretic consequences. Using equations (1) and (2), the decision function for classifying data examples (regardless whether they belong to the training or a test set) simply becomes

$$F(\boldsymbol{x}) = sign\big(\langle \boldsymbol{w},\boldsymbol{x} \rangle + b \big) = sign\big(\sum_i (\alpha_i \, y_i \; \langle \boldsymbol{x},\boldsymbol{x}_i \rangle) + b \big) \qquad (3).$$

The argument of the sign-function can be regarded as a measure for the classification confidence. From now on, we will refer to it as the *discriminant value*. In the context of the binary classification problem, its sign indicates the predicted class membership of a data point while its absolute value is roughly correlated with the unambiguousness of this decision. (Note, however, that abnormally large numbers might be pointing to the membership in a different class that was not considered when training was carried out.)

In order to handle linearly non-separable problems, the simple SVM algorithm sketched above can be extended in two ways. First, a soft margin approach is applied to provide some tolerance for a limited number of training errors (i.e. data points lying at the wrong side of the separating hyperplane). This can be implemented in different ways, but the common principle is always to introduce additional parameter(s) that can control the trade-off between training accuracy and the size of the margin, from which the latter one is closely related to the

generalization performance of a classifier. A suitable choice for the values of these controlling parameters can be done either by estimating the amount of noise in the data or by assessing the classification performance with independent test data. Secondly, all training patterns can be mapped to a higher dimensional space ("feature space") prior to constructing the optimal separating hyperplane. By using a nonlinear mapping, data which is not linearly separable in the original input space can acquire this attractive property in the feature space. Surprisingly, there is a way in practice to avoid the computationally expensive mapping transformation altogether. This is possible since there is a formulation of the classification problem that is based solely on dot products between mapped data points. Therefore, real valued *kernel functions* $k(\mathbf{x}_i, \mathbf{x}_j)$ that represent the inner product carried out in the higher dimensional feature space can be introduced in order to replace the dot product between vectors of the input space. The decision function (3) therefore can be written in the form

$$F(\mathbf{x}) = sign\left(\sum_i (\alpha_i y_i k(\mathbf{x},\mathbf{x}_i)) + b\right) \quad (4).$$

From this representation it can be seen that kernel functions can also be interpreted as (nonlinear) pairwise similarity measures. Summarizing, the "kernel trick" allows to combine the mathematical elegance of linear decision functions with the power of dealing with problems which are not linearly separable.

Although being a binary classification technique by its very nature, the SVM approach can be applied to multiple class problems as well since in such a situation it is always possible to break down the principal classification task into several binary decisions. One possibility to do so is the One-versus-All (OVA) training method. Following the OVA protocol, n single SVM classifiers have to be generated if there are n different classes, each of them discriminating between one class (containing the positive examples) and the combined set of examples from all other classes (acting as negative examples). Classification of a new pattern

then involves combining the output of n SVMs and making a decision based on the resulting set of discriminant values.

Three dimensional data visualization. There are some commonly used methods to present the classification results obtained from SVMs. One option is to directly show the class predictions as calculated from the decision function (equation 4) or the respective discriminant values. Alternatively, it is possible to estimate the probabilities of class assignments based on a distribution analysis of the discriminant values. Sigmoid functions are often used to build such a model. These methods provide a well-defined and compact "one dimensional" measure for the classification of a single test example. On the other hand, much information about the internal structure of the data is lost since from the discriminant values alone it is not possible to determine the contribution of individual features to the score and to identify subsets of training or test examples that share a similar distribution of feature values and are therefore closely related.

In order to retrieve at least some of this information, we have tried and hereby propose the following method that leads to mapping of all data examples into a three dimensional space. Our approach is to split up the dot product $\langle \mathbf{w},\mathbf{x} \rangle$ in the decision function of the linear kernel (equation 2) and define a mapping $\mathbf{\Phi}$,

$$\boldsymbol{\Phi}(\mathbf{x}) = \sum_{i\in I} (w_i \cdot x_i)\, \mathbf{e}_x + \sum_{i\in J} (w_i \cdot x_i)\, \mathbf{e}_y + \sum_{i\in K} (w_i \cdot x_i)\, \mathbf{e}_z \qquad (5)$$

where I, J and K are disjoint sets of indices whose union is $\{1, 2,\ldots, m\}$, i.e. the complete set of indices for the m features which are known for each training example and $\mathbf{e}_x$, $\mathbf{e}_y$, $\mathbf{e}_z$ are orthogonal unit vectors spanning a Cartesian coordinate system. This transformation splits one discriminant value into three components, whereby each component is given as a linear combination of different features. By inserting the feature weights w_i obtained from one binary SVM classifier and plotting $\boldsymbol{\Phi}(\mathbf{x})$ for all training patterns in a three dimensional scatter plot, it is possible to project down a potentially very high dimensional separation problem into

a three dimensional cube while preserving the separability information together with some inherent structure of the data. For example, in the case of linear separable data there exists also (at least) one two dimensional plane in the projected data set that separates positive from negative examples. Of course there are many possible ways to distribute the features among the sets *I*, *J* and *K*. As a practical approach for standardizing the mapping, we first sort the features by their absolute weights $|w_i|$ and split the resulting list in three equal parts, each containing exactly one third of all features (barring rounding effects). Therefore the "most important" 33% of the features are collected in set *I*, while for example the least important ones are gathered in subset *K*. This means that the components of $\boldsymbol{\Phi(x)}$ are not equivalent. Often there are many features that are completely redundant for the classification (as it is certainly the case for microarray experiments) and the data is completely separable with the $\boldsymbol{e}_x$ component alone. However, according to our experience with real microarray data it is often the case that subclusters of microarrays (representing for example slight differences in the treatment of individual samples or the specific effect of compounds that have been put together in one class) can be observed when all components are considered simultaneously. A visual inspection of the three dimensional mapping (5) can therefore lead to a deeper understanding of the data set and the detection of unknown subgroups or single outliers. Furthermore, this transformation can also be carried out with the test examples so that all data can be shown in a single scatter plot. It is then possible to compare the distribution of the test data with those of different training groups in order to detect similarities and dissimilarities among the groups. We use this method routinely to explore our data sets.

Sample Preparation and Hybridization

Briefly, total RNA was isolated using RNAzol (Tel-Test Inc., Friendswood, TX) and the commercially available kit Bio101 (Qbiogene, Inc., Carlsbad, CA). Total RNA was purified

using RNeasy columns (Qiagen, Basel, Switzerland). RNA integrity was assessed on an agarose gels or with a Bioanalyzer 2100 (Agilent Technologies, Palo Alto, CA). Double-stranded cDNA was synthesized from total RNA (usually 20µg) using a commercially available kit (Superscript Choice system, Life Technologies, Basel, Switzerland) and an oligo $(dT)_{24}$VN-anchored T7 primer (HPLC purified, Microsynth, Balgach, Switzerland). Biotinylated cRNA was synthesized from cDNA using the Megascript kit (Ambion, Austin, TX) and biotinylated nucleotides (Bio-11-CTP and Bio-16-UTP, Roche Molecular Biosciences, Mannheim, Germany). In vitro transcription products were purified on RNeasy columns (Qiagen, Basel, Switzerland), the cRNA was fragmented in a solution of 40 mM Tris-acetate, pH 8.1, 100mM KOAc, and 30 mM MgOAc at 94ºC for 35 minutes and fragmentation was assessed by agarose gel electrophoresis or with a Bioanalyzer 2100 (Agilent Technologies, Palo Alto, CA). Hybridization to rat-specific microarrays RG-U34A (Affymetrix, Santa Clara, CA) was carried out according to manufacturer's instructions overnight at 45ºC. Microarrays were washed and stained on the GeneChip Fluidics Station 400 from Affymetrix as described in the manufacturer's instructions (R-Phycoerythrin Streptavidin was from Molecular Probes, Eugene, OR; Goat IgG was from SIGMA, Buchs, Switzerland; biotinylated Goat anti-streptavidin was fromVector Labs, Burlingame, CA).

<u>Supplemental Data Table 1:</u> Time-dependent genes

**Affymetrix ID**	**Description**
AF086624_s_at	serine threonine kinase pim3
AF089825_at	activin beta e
L32601_s_at	20 alpha-hydroxysteroid dehydrogenase
M25804_at	nuclear receptor subfamily 1, group d, member 1
M35826cds_s_at	rat mitochondrial nadh-dehydrogenase
M67465_at	hydroxy-delta-5-steroid dehydrogenase, 3 beta- and steroid delta-isomerase
M76767_s_at	fatty acid synthase
M90514mRNA_at	ratdrohom rattus rattus dna fragment homologous to drosophila pecanex locus
M95591_at	farnesyl diphosphate farnesyl transferase 1
rc_AA799616_at	transcribed sequence with moderate similarity to protein sp:p53801
rc_AA799724_g_at	transcribed sequence with strong similarity to protein sp:p97304
rc_AA817964_s_at	paraoxonase 1
rc_AA866321_at	transcribed sequences
rc_AI175935_at	myosin ie

Supplemental Data Table 2a: Profiles used for testing SVM models and calculating test MCC

**Treatment**	**Vehicle/ Route**	**Expected Binary class/ 4-MOT class**	**Liver Histopathology**	**Serum Clinical Chemistry**
1,2-dichlorobenzene (1500 mmol/kg, 24 h) [CAS No. 95-50-1, Fluka]	Corn oil/i.p.	Toxic/Direct	Centrilobular to midzonal hepatocellular hydropic swelling with necrosis and inflammation, glycogen depletion	Increased ALP
Rx99 (400 mg/kg/day, 24 h) 5-HT6 antagonist [CAS-No. not available, Roche]	H_2O/ p.o.	Toxic/Steatotic	Fatty change	Increased glucose
Rx99 (100 mg/kg/day, 7 d) 5-HT6 antagonist [CAS-No. not available, Roche]	H_2O/ p.o.	Toxic/Steatotic	Fatty change	Increased glucose
Amineptine (0.25 mmol/kg/day, 4 d) [CAS No. 57574-09-1, Servier Laboratories]	Saline/i.p.	Toxic/Steatotic	Fatty change, glycogen depletion	Increased GGT; decreased bilirubin, triglycerides, A/G ratio
Amineptine (0.125 mmol/kg/day, 4 d) [CAS No. 57574-09-1, Servier Laboratories]	Saline/i.p.	Toxic/Steatotic	Fatty change, glycogen depletion	Decreased bilirubin, triglycerides, A/G ratio
ANIT (150 mg/kg, 6 h) [CAS-No	Corn	Toxic/Cholestatic	NSF	Increased bilirubin, glucose

551-06-4, Sigma]	oil/p.o.			
APAP (1000 mg/kg, 24h) [CAS-No 103-90-2, Fluka]	Saline + 0.5% DMSO/ p.o.	Toxic/Direct	Centrilobular hepatocellular vacuolation, single cell necrosis, polymorphonuclear infiltration	Decreased triglycerides
Bromobenzene (1 mmol/kg, 24 h) [CAS No. 108-86-1, Aldrich]	Corn oil/i.p.	Toxic/Direct	Centrilobular to midzonal hydropic swelling, necrosis with mixed inflammation, glycogen depletion	Increased ALP, 5'-NT
CCl_4 (0.25 mg/kg, 24h) [CAS No. 56-23-5, Fluka]	Corn oil/p.o.	Toxic/Direct	Hepatocellular degeneration, single cell necrosis with inflammation, microvesicular steatosis	Increased GGT
Rx10 (125 mg/kg/day, 5 d) antidiabetic [CAS-No. not available, Roche]	Klucel/p.o.	Toxic/Steatotic	NSF[a]	ND[a]
Lithocholic acid (0.12 mmol/kg, 6 h) [CAS-No 434-13-9, Fluka]	3.5% albumin in saline/i.v.	Toxic/Cholestatic	ND	Increased bilirubin (<6h), triglycerides
Methylene Dianiline (100 mg/kg, 3 h) [CAS No. 101-77-9, Fluka]	Corn oil/p.o.	Toxic/Cholestatic	Single cell necrosis of bile duct epithelium	Increased glucose, total bilirubin, bile acids; decreased total protein
Rx50 (1.0 mg/kg/day, 14 d) PPAR-α,γ coagonist [CAS-No. not available, Roche]	PBS/p.o.	Toxic/Perox. Prolif.	Increased liver weight; slight hepatocellular hypertrophy, infiltration,	Decreased protein
Rx51 (0.13 mg/kg/day, 14 d) PPAR-	PBS/p.o.	Toxic/Perox. Prolif.	Increased liver weight; minimal hepatocellular	Decreased protein, cholesterol

α,γ coagonist [CAS-No. not available, Roche]			hypertrophy, mixed infiltration	
Rx60 (0.38 mg/kg/day, 14 d) [CAS-No. not available, Roche]	PBS/p.o.	Toxic/Perox. Prolif.	Slight hepatocellular hypertrophy, mixed infiltration, single cell necrosis in some rats	Decreased bilirubin
Rx90 (60 mg/kg/day, 14 d) PPAR-δ agonist [CAS-No. not available, Roche]	PBS/p.o.	Toxic/Perox. Prolif.	Diffuse hepatocellular hypertrophy, occasional liver enlargement	NSF
Rx90 (300 mg/kg/day, 7 d) PPAR-δ agonist [CAS-No. not available, Roche]	PBS/p.o.	Toxic/Perox. Prolif.	Diffuse hepatocellular hypertrophy, occasional liver enlargement	Increased bilirubin, glucose, triglycerides, cholesterol, protein, ALT, ALP
17a-ethinylestradiol (5 mg/kg, 6 h) [CAS-No 57-63-6, Sigma]	Propylene glycol	Toxic/Cholestatic	Mild glycogen depletion in a few rats	Decreased cholesterol, triglycerides
D-Galactosamine (400 mg/kg, 24 h) [CAS-No 7535-00-4, Sigma]	Saline/i.p.	Toxic/Direct	Diffuse hepatocellular hypertrophy, apoptosis, necrosis, inflammation, glycogen depletion; bile duct proliferation, oval cell proliferation	Increased ALT, AST
Glibenclamide (2.5 mg/kg, 6 h) [CAS No. 10238-21-8, Roche]	7.5% gelatine i.v.	Toxic/Cholestatic	NSF	Decreased glucose

Supplemental Data Table 2b: Profiles used for assessing SVM models (No MCC calculation)

Treatment	Vehicle/ Route	Expected Binary class/ 4-MOT class	Liver Histopathology	Serum Clinical Chemistry
Phenobarbital (80 mg/kg/day, 6 h) [CAS No. 50-06-6, Fluka]	Saline/i.p.	Toxic/Other	Centrilobular hepatocellular hypertrophy	NSF
Phenobarbital (80 mg/kg/day, 24 h) [CAS No. 50-06-6, Fluka]	Saline/i.p.	Toxic/Other	Centrilobular hepatocellular hypertrophy	NSF
Gentamicin (100 mg/kg/day, 24 h) [1403-66-3, Sigma]	Saline/s.c.	Non-toxic	NSF	Increased GGT, glucose; decreased triglycerides
Lazabemide (1 g/kg/day, 4 d) [CAS No. 103878-84-8, Roche]	SSV/p.o.	Non-toxic	NSF	Increased albumin
L-deprenyl (20 mg/kg/day, 4 d) [CAS No. 14611-52-0,Sigma]	SSV/p.o.	Non-toxic	NSF	Increased glucose, SDH, GGT
LPS (4 mg/kg, 6 h)[Sigma, L2887 E. Coli serotype 0128-B12]	Saline/i.v.	Toxic/Other	Glycogen depletion, sinusoidal granulocytosis	Increased GLD; decreased glucose, total protein, albumin
LPS (4 mg/kg, 24 h) [Sigma, L2887 E. Coli serotype 0128-B12]	Saline/i.v.	Toxic/Other	Glycogen depletion, sinusoidal granulocytosis	Increased bilirubin, ALP, SDH,; decreased triglycerides, albumin
Indomethacin (20 mg/kg, 6 h) [CAS No. 53-86-1, Sigma]	Saline/p.o.	Toxic/Several	Mild hepatocellular hypertrophy, glycogen depletion	Decreased A/G ratio

Indomethacin (20 mg/kg, 24 h) [CAS No. 53-86-1, Sigma]	Saline/p.o.	Toxic/Several	Mild hepatocellular hypertrophy, glycogen depletion	Decreased total protein, albumin
Indomethacin (5 mg/kg/day , 7 d) [CAS No. 53-86-1, Sigma]	Saline/p.o.	Toxic/Several	Mild hepatocellular hypertrophy, glycogen depletion	Increased AST, LDH, ALP, GGT, triglycerides; decreased glucose, total protein, albumin

[a] Clinical chemistry and histopathological evaluation were not performed on the animals used for gene profiling. Repeated dosing of rats in other studies showed no fatty change, but hepatic steatosis was seen in dogs treated with this antidiabetic compound. *In vitro* treatment of rat primary hepatocytes indicated steatotic potential by showing inhibition of β-oxidation and fat accumulation.

ND. Not done; NSF. No significant findings

Toxicogenomics QC Guidelines

Supplemental Data Table 3a: Microarray Quality

Parameter	Acceptable Value	Rationale
Sum of average difference (SAD)	<5 million	Very low SAD results in signals from low abundancy transcripts being lost in the background. Very high signals result in signal saturation for highly abundant transcripts. Microarrays for one study should be as consistent as possible to avoid large Normalisation factors.
Percent Present	>37%	Good global measure of data quality. Correlates with SAD.
3'/5' ratio	<3	Flag for poor quality mRNA/cRNA.
Raw Q (noise)	<3	Measure of microarray performance.

Supplemental Data Table 3b: Parameters and settings for analyzing studies (individual compounds)

Parameter	Value	Rationale
Normalization factor for each microarray	0.5 to 2	Large Normalisation factors are due to large differences in Sum of Average Difference between microarrays in a set. This is more likely to result in Change Factors that have poor statistical quality and which are therefore uninterpretable.
Adjust Avg_diff	4	Avoids generation of extremely high Change Factors for genes with negative Average Difference.
Nalimov Outlier Exclusion	99%	Improves statistical reliability of Change Factors

These values are applicable for a scanner setting of 1800.

Supplemental Data Table 4: Feature list for the Multi-Toxicity Model (ν)

Direct acting

**Affymetrix ID**	**Description**
AB005547_at	aquaporin 8
AB013732_at	udp-glucose dehydrogeanse
AF010597_s_at	atp-binding cassette, sub-family b (mdr/tap), member 11
AF040954_at	putative protein phosphatase 1 nuclear targeting subunit
AF045464_s_at	aflatoxin b1 aldehyde reductase
C07012_f_at	peptidylprolyl isomerase c-associated protein
D17809_at	beta-4n-acetylgalactosaminyltransferase
D25224_at	laminin receptor 1 (67kd, ribosomal protein sa)
D25224_g_at	laminin receptor 1 (67kd, ribosomal protein sa)
D28339_s_at	3-hydroxyanthranilate 3,4-dioxygenase
D38061exon_s_at	d38061exon ratugt1a1a rat dna for udp glucuronosyltransferase, exon 1
D44494_at	3-hydroxyanthranilate 3,4-dioxygenase
D45247_g_at	ratpsrcx rat mrna for proteasome subunit rcx, complete cds
D86580_at	nuclear receptor subfamily 0, group b, member 2
J00728cds_f_at	cytochrome p450, 2b19
J02657_s_at	cytochrome p450, subfamily iic (mephenytoin 4-hydroxylase)
J02679_s_at	nad(p)h dehydrogenase, quinone 1
J02861mRNA_s_at	cytochrome p450 2c13
J03588_at	guanidinoacetate methyltransferase
J05122_at	benzodiazepin receptor (peripheral)
K01721mRNA_s_at	cytochrome p450, 2b19
L15079mRNA_s_at	atp-binding cassette, sub-family b (mdr/tap), member 4
L16764_s_at	heat shock 70kd protein 1a
L22339_g_at	sulfotransferase family 1a, phenol-preferring, member 2
L25331_at	procollagen-lysine, 2-oxoglutarate 5-dioxygenase (lysine hydroxylase, ehlers-danlos syndrome type vi)

L81136cds_f_at	l81136cds ratrps2r1a rattus norvegicus (strain r21) rps2r1 preliminary dna, complete cds
M11794cds#2_f_at	metallothionein
M25804_g_at	nuclear receptor subfamily 1, group d, member 1
M58041_s_at	cytochrome p450 2c22
M63282_at	activating transcription factor 3
M74067_at	claudin 3
M76704_s_at	o6-methylguanine-dna methyltranferase
M81855_at	p-glycoprotein/multidrug resistance 1
M82855cds_s_at	cytochrome p450 2c13
M91235_f_at	rat vl30 element mrna
M95762_at	solute carrier family 6 (neurotransmitter transporter, gaba), member 13
rc_AA685974_at	rattus norvegicus transcribed sequence with weak similarity to protein ref:np_116187.1 (h.sapiens) hypothetical protein flj14503 [homo sapiens]
rc_AA799899_i_at	rattus norvegicus transcribed sequence with strong similarity to protein ref:np_000971.1 (h.sapiens) ribosomal protein l18a; 60s ribosomal protein l18a [homo sapiens]
rc_AA858607_at	rattus norvegicus transcribed sequence with moderate similarity to protein ref:np_003842.1 (h.sapiens) cellular repressor of e1a-stimulated genes [homo sapiens]
rc_AA858673_at	pancreatic secretory trypsin inhibitor type ii (psti-ii)
rc_AA891286_at	thioredoxin reductase 1
rc_AA891737_at	rattus norvegicus transcribed sequences
rc_AA891769_at	rattus norvegicus focal adhesion kinase (fak) mrna, alternative 5`utr
rc_AA892353_at	rattus norvegicus transcribed sequence with moderate similarity to protein ref:np_078863.1 (h.sapiens) hypothetical protein flj22353 [homo sapiens]
rc_AA892797_at	phosphoglycerate kinase 1
rc_AA893246_at	rattus norvegicus transcribed sequence with strong similarity to protein sp:q9y5k8 (h.sapiens) vatd_human vacuolar atp synthase subunit d (v-atpase d subunit) (vacuolar proton pump d subunit) (v-atpase 28 kda accessory protein)
rc_AA893495_at	rattus norvegicus transcribed sequence with weak similarity to protein sp:p08185 (h.sapiens) cbg_human corticosteroid-binding globulin precursor (cbg) (transcortin)
rc_AA942685_at	cytosolic cysteine dioxygenase 1

rc_AA945082_at	glutathione-s-transferase, alpha type2
rc_AA945583_at	hydroxysteroid (17-beta) dehydrogenase 10
rc_AA945704_at	rattus norvegicus transcribed sequences
rc_AA945806_at	ribosomal protein s14
rc_AA963449_s_at	cytochrome p450, subfamily 51
rc_AI009806_at	dynein, cytoplasmic, light chain 1
rc_AI102562_at	metallothionein
rc_AI103074_at	ribosomal protein s12
rc_AI104399_at	triosephosphate isomerase 1
rc_AI145931_at	udp-n-acetylglucosamine-2-epimerase/n-acetylmannosamine kinase
rc_AI171562_at	nuclear protein e3-3
rc_AI172293_at	sterol-c4-methyl oxidase-like
rc_AI172452_at	rattus norvegicus transcribed sequence with moderate similarity to protein ref:np_004709.2 (h.sapiens) cytochrome c oxidase subunit viia polypeptide 2 like; cytochrome c oxidase subunit vii-related protein; estrogen receptor binding cpg island; cytochrome c oxidase polypeptide viia-heart [homo sapiens]
rc_AI176456_at	rattus norvegicus transcribed sequence with moderate similarity to protein sp:p04732 (h.sapiens) mt1e_human metallothionein-ie (mt-1e)
rc_AI177096_at	rattus norvegicus transcribed sequence with moderate similarity to protein pir:rthua (h.sapiens) rthua adenine phosphoribosyltransferase (ec 2.4.2.7) - human
rc_AI177366_at	integrin, beta 1
rc_AI178135_at	complement component 1, q subcomponent binding protein
rc_AI231807_at	ferritin light chain 1
rc_AI231807_g_at	ferritin light chain 1
rc_AI233261_i_at	glutamate cysteine ligase, modifier subunit
rc_AI639246_at	rattus norvegicus transcribed sequence with moderate similarity to protein ref:np_006682.1 (h.sapiens) extracellular link domain-containing 1; lymphatic vessel endothelial hyaluronan receptor 1; hyaluronic acid receptor [homo sapiens]
rc_AI639488_at	rattus norvegicus transcribed sequence with moderate similarity to protein prf:1814460a (h.sapiens) 1814460a p53-associated protein [homo sapiens]

rc_H33491_at	phenylalkylamine ca2+ antagonist (emopamil) binding protein
S71021_s_at	malignancy-related c140 product [rats, thyroid frtl-tc cells, mrna partial, 746 nt]
U04733_s_at	arachidonic acid epoxygenase
U25264_at	selenoprotein w muscle 1
U32314_at	pyruvate carboxylase
U32681_at	deleted in malignant brain tumors 1
U32681_g_at	deleted in malignant brain tumors 1
U36992_at	cytochrome p450, subfamily 7b, polypeptide 1
U39943_s_at	cytochrome p450 monooxygenase
U55938_at	sialyltransferase 8 c
U63923_at	thioredoxin reductase 1
U88036_at	solute carrier family 21 (organic anion transporter), member 5
V01235_at	fatty acid binding protein 1, liver
X02610_g_at	enolase 1, alpha
X04229cds_s_at	glutathione s-transferase, mu 1
X06423_at	ribosomal protein s8
X06483cds_at	ribosomal protein l32
X06916_at	s100 calcium-binding protein a4
X14181cds_s_at	x14181cds rrrpl18a rat mrna for ribosomal protein l18a
X15096cds_s_at	acidic ribosomal protein p0
X15580complete_seq_s_at	6-phosphofructo-2-kinase/fructose-2,6-biphosphatase 1
X55153mRNA_s_at	x55153mrna rrrp2g r.rattus rp2 gene for ribosomal protein p2
X57432cds_s_at	ribosomal protein s2
X58294_at	carbonic anhydrase 2
X58389cds_s_at	rat amino acid starvation-induced protein mrna, 3` end
X59859_i_at	decorin
X62145cds_g_at	x62145cds rrrpl8 r.rattus mrna for ribosomal protein l8
X74593_at	sorbitol dehydrogenase
X89225cds_s_at	solute carrier family 3, member 2
X94242_at	ribosomal protein l14

X96437mRNA_g_at	x96437mrna rnprg1 r.norvegicus prg1 gene

Peroxisomals proliferators/PPAR agonists

**Affymetrix ID**	**Description**
J02749_at	acetyl-coenzyme a acyltransferase 1 (peroxisomal 3-oxoacyl-coenzyme a thiolase)
J02749_g_at	acetyl-coenzyme a acyltransferase 1 (peroxisomal 3-oxoacyl-coenzyme a thiolase)
M14972_i_at	cytochrome p450,4a1
rc_AA924267_s_at	cytochrome p450,4a1

Cholestasis

**Affymetrix ID**	**Description**
AB011369_s_at	protein kinase c-binding protein beta15
AB017544_at	peroxisomal biogenesis factor 14
D89983_at	ornithine decarboxylase antizyme inhibitor
L27843_s_at	protein tyrosine phosphatase 4a1
L81136cds_f_at	l81136cds ratrps2r1a rattus norvegicus (strain r21) rps2r1 preliminary dna, complete cds
M37828_at	cytochrome p450, 4a12
M58758_g_at	atpase, h+ transporting, lysosomal noncatalytic accessory protein 1a
M75281_at	rat cystatin s (cyss) gene, complete cds /cds=(73,498) /gb=m75281 /gi=294537 /ug=rn.10908 /len=710
M83143_g_at	sialyltransferase 1 (beta-galactoside alpha-2,6-sialytransferase)
rc_AA891769_at	rattus norvegicus focal adhesion kinase (fak) mrna, alternative 5`utr
rc_AA891812_g_at	adducin 1, alpha
rc_AA892400_at	rattus norvegicus transcribed sequence with moderate similarity to protein ref:np_057084.1 (h.sapiens) cgi-47 protein; mitochondrial cca-adding trna-nucleotidyltransferase [homo sapiens]
rc_AA893485_at	est197288 rattus norvegicus cdna, 3` end /clone=rliad06 /clone_end=3` /gb=aa893485 /gi=3020364 /ug=rn.4088 /len=434
rc_AA945143_at	tryptophan 2,3-dioxygenase
S85184_g_at	cyclic protein-2=cathepsin l proenzyme [rats, sertoli cells, mrna, 1790 nt]

U05784_s_at	microtubule-associated proteins 1a/1b light chain 3
X15580complete_seq_s_at	6-phosphofructo-2-kinase/fructose-2,6-biphosphatase 1
X63594cds_at	x63594cds rrrlif1 r.rattus rl/if-1 mrna
X68199_at	myosin ib

Steatosis

**Affymetrix_ID**	**Description**
AF016503_s_at	procollagen c-proteinase enhancer protein
D10354_s_at	glutamic-pyruvate transaminase (alanine aminotransferase)
D13912_s_at	cytochrome p450, subfamily 3a, polypeptide 3
D21132_at	phosphotidylinositol transfer protein, beta
D50580_at	phenobarbital-inducible carboxylesterase (liver)
D89655_at	scavenger receptor class b, member 1
J00728cds_f_at	cytochrome p450, 2b19
K00996mRNA_s_at	cytochrome p450, 2b19
L24207_i_at	cytochrome p450, subfamily 3a, polypeptide 3
L24207_r_at	cytochrome p450, subfamily 3a, polypeptide 3
L32132_at	lipopolysaccharide binding protein
M11251cds_f_at	cytochrome p450, 2b19
M13234cds_f_at	cytochrome p450, 2b19
M14776_f_at	ratcy45ab rat cytochrome p-450 pb-1 mrna, partial cds
M23566exon_s_at	m23566exon rata2mac2 rattus norvegicus alpha-2-macroglobulin gene, 3` end
M24239cds#2_f_at	cytochrome p450, 2c37
M64733mRNA_s_at	m64733mrna rattrpm2b rat trpm-2 gene, complete cds
rc_AA800849_f_at	est190346 rattus norvegicus cdna, 3` end /clone=atcc-2008268 /clone_end=3` /gb=aa800849 /gi=2863804 /ug=rn.7381 /len=335
rc_AA875121_at	nuclear transcription factor-y gamma
rc_AA894200_g_at	rattus norvegicus transcribed sequence with strong similarity to protein sp:p16475 (h.sapiens) mlen_human myosin light chain alkali, non-muscle isoform (mlc3nm) (lc17a)

	(lc17-nm) (smooth muscle myosin alkali light chain) (nonmuscle myosin light chain 3) (mlc-3)
rc_AA945571_s_at	cytochrome p450, subfamily iic6
rc_AA946503_at	lipocalin 2
rc_AI237016_at	h2a histone family, member y
U33500_at	rattus norvegicus retinol dehydrogenase type ii mrna, complete cds
U88036_at	solute carrier family 21 (organic anion transporter), member 5
X51529_at	phospholipase a2, group iia (platelets, synovial fluid)
X62086mRNA_s_at	x62086mrna rncyp3a1 r.norvegicus cyp3a1 gene for cytochrome p450 pcn1
X62146cds_at	x62146cds rrrpl11 r.rattus mrna for ribosomal protein l11

Controls

**Affymetrix ID**	**Description**
AB009463_g_at	low density lipoprotein receptor-related protein 3
AB010635_s_at	carboxylesterase 2 (intestine, liver)
AB012230_g_at	nuclear factor i/b
AF006617_at	stress 70 protein chaperone, microsome-associated, 60kd human homolog
AF009604_at	sh3 domain protein 2 c1
AF015304_at	solute carrier family 29, member 1
AF017437_at	integrin-associated protein
AF022774_g_at	rabphilin 3a-like (without c2 domains)
AF023087_s_at	early growth response 1
AF025671_s_at	caspase 2
AF045464_s_at	aflatoxin b1 aldehyde reductase
AF080507_at	rattus sp. mannose-binding protein mrna, partial cds
AF081148_s_at	calcium-independent alpha-latrotoxin receptor homolog 2
AF090134_at	lin-7-ba
AF102804_s_at	adenosine a3 receptor
AJ006064_g_at	coronin, actin-binding protein, 1b
D00752_at	liver regeneration protein lrryan

D10026_s_at	glutathione s-transferase, theta 2
D12769_g_at	kruppel-like factor 9
D13907_g_at	mitochondrial processing peptidase beta
D26154UTR#1_g_at	d26154utr#1 ratrb109 rat mrna for rb109 (brain specific protein), complete cds
D29766Poly_A_Site#1_at	v-crk-associated tyrosine kinase substrate
D37934_g_at	rat mrna for 5e5 antigen, complete cds /cds=(765,3242) /gb=d37934 /gi=531260 /ug=rn.3196 /len=4492
D43964_at	bile acid-coenzyme a: amino acid n-acyltransferase
D49785_at	mitogen activated protein kinase kinase kinase 12
D85189_at	fatty acid coenzyme a ligase, long chain 4
D89655_at	scavenger receptor class b, member 1
E01524cds_s_at	p450 (cytochrome) oxidoreductase
J00728cds_f_at	cytochrome p450, 2b19
J02596cds_at	j02596cds ratapoa02 rat apolipoprotein c-iii gene, complete cds
J02657_s_at	cytochrome p450, subfamily iic (mephenytoin 4-hydroxylase)
J02679_s_at	nad(p)h dehydrogenase, quinone 1
J02749_at	acetyl-coenzyme a acyltransferase 1 (peroxisomal 3-oxoacyl-coenzyme a thiolase)
J03572_i_at	alkaline phosphatase, tissue-nonspecific
J03583_at	clathrin, heavy polypeptide (hc)
J04171_at	glutamate oxaloacetate transaminase 1
K00996mRNA_s_at	cytochrome p450, 2b19
K01721mRNA_s_at	cytochrome p450, 2b19
L00320cds_f_at	cytochrome p450, 2b19
L15079mRNA_s_at	atp-binding cassette, sub-family b (mdr/tap), member 4
L24207_i_at	cytochrome p450, subfamily 3a, polypeptide 3
L26268_at	b-cell translocation gene 1
L27843_s_at	protein tyrosine phosphatase 4a1
L32132_at	lipopolysaccharide binding protein
M10068mRNA_s_at	p450 (cytochrome) oxidoreductase
M10934_s_at	ratrbpa rat retinol-binding protein (rbp) mrna, partial cds

M11251cds_f_at	cytochrome p450, 2b19
M12822cds_f_at	m12822cds ratigkcaa rat (r.leucopus cooktownensis) ig germline kappa-chain gene c-region, 3` end
M13234cds_f_at	cytochrome p450, 2b19
M18363cds_s_at	cytochrome p450, subfamily iic (mephenytoin 4-hydroxylase)
M18416_at	early growth response 1
M20629_s_at	esterase 2
M23566exon_s_at	m23566exon rata2mac2 rattus norvegicus alpha-2-macroglobulin gene, 3` end
M26125_at	epoxide hydrolase 1
M31837_at	insulin-like growth factor binding protein 3
M32783cds_i_at	m32783cds ratenka3 rat dynorphin gene, exon 3
M35601_g_at	fibrinogen, a alpha polypeptide
M37482_at	inhibin beta-a
M55532_at	kupffer cell receptor
M57507_at	guanylate cyclase, soluble, beta 2
M58758_g_at	atpase, h+ transporting, lysosomal noncatalytic accessory protein 1a
M60103_at	protein tyrosine phosphatase, receptor type, f
M60655_at	adrenergic receptor, alpha 1b
M74067_at	claudin 3
M86912exon_g_at	m86912exon ratat1b rat angiotensin receptor (at1) gene, single exon
M91599mRNA_g_at	m91599mrna ratfgr4a rat fibroblast growth factor receptor subtype 4 (fgfr4) mrna, complete cds
rc_AA799616_at	rattus norvegicus transcribed sequence with moderate similarity to protein sp:p53801 (h.sapiens) pttg_human pituitary tumor-transforming gene 1 protein-interacting protein (pituitary tumor-transforming gene protein binding factor) (pttg-binding factor) (pbf)
rc_AA800626_at	rattus norvegicus transcribed sequences
rc_AA818122_f_at	sulfotransferase, hydroxysteroid preferring 2
rc_AA858573_s_at	spp-24 precursor
rc_AA875598_at	rattus norvegicus transcribed sequence with strong similarity to protein sp:q13617 (h.sapiens) cul2_human cullin homolog 2 (cul-2)

rc_AA891220_at	rattus norvegicus transcribed sequences
rc_AA891740_at	thymic stromal-derived lymphopoietin, receptor
rc_AA891842_g_at	rattus norvegicus transcribed sequence with weak similarity to protein ref:np_057713.1 (h.sapiens) hypothetical protein loc51323 [homo sapiens]
rc_AA892287_at	rattus norvegicus transcribed sequence with weak similarity to protein ref:np_061123.2 (h.sapiens) g protein-coupled receptor, family c, group 5, member c, isoform b, precursor; orphan g-protein coupled receptor; retinoic acid inducible gene 3 protein; retinoic acid responsive gene protein [homo sapiens]
rc_AA892297_at	histone deacetylase 2
rc_AA892380_at	rattus norvegicus transcribed sequence with moderate similarity to protein ref:np_006406.1 (h.sapiens) serine palmitoyltransferase, long chain base subunit 1; serine palmitoyltransferase subunit i [homo sapiens]
rc_AA892388_at	rattus norvegicus transcribed sequence with moderate similarity to protein ref:np_055141.2 (h.sapiens) death-associated protein kinase 2 [homo sapiens]
rc_AA892417_at	ephrin a1
rc_AA892522_at	rattus norvegicus transcribed sequences
rc_AA892768_at	rattus norvegicus transcribed sequence with strong similarity to protein ref:np_055268.1 (h.sapiens) putative breast adenocarcinoma marker (32kd) [homo sapiens]
rc_AA893035_s_at	hp33
rc_AA893485_at	est197288 rattus norvegicus cdna, 3` end /clone=rliad06 /clone_end=3` /gb=aa893485 /gi=3020364 /ug=rn.4088 /len=434
rc_AA893495_at	rattus norvegicus transcribed sequence with weak similarity to protein sp:p08185 (h.sapiens) cbg_human corticosteroid-binding globulin precursor (cbg) (transcortin)
rc_AA893552_at	kallistatin
rc_AA894090_at	rattus norvegicus transcribed sequence with strong similarity to protein sp:o43808 (h.sapiens) pm34_human peroxisomal membrane protein pmp34 (34 kda peroxisomal membrane protein) (solute carrier family 25, member 17)
rc_AA894258_at	ubiquitin-conjugating enzyme e2d 3 (homologous to yeast ubc4/5)
rc_AA900582_at	alpha-2-macroglobulin
rc_AA945050_f_at	rat senescence marker protein 2a gene, exons 1 and 2

rc_AA945082_at	glutathione-s-transferase, alpha type2
rc_AA945143_at	tryptophan 2,3-dioxygenase
rc_AA945321_at	albumin
rc_AA946503_at	lipocalin 2
rc_AI008815_s_at	cytochrome c, somatic
rc_AI013472_at	er transmembrane protein dri 42
rc_AI102562_at	metallothionein
rc_AI137856_s_at	p450 (cytochrome) oxidoreductase
rc_AI175764_s_at	stearoyl-coenzyme a desaturase 1
rc_AI177366_at	integrin, beta 1
rc_AI229655_at	rattus norvegicus transcribed sequences
rc_AI232087_at	hydroxyacid oxidase (glycolate oxidase) 3
rc_AI232256_at	cytochrome b5, outer mitochondrial membrane isoform
rc_AI233173_at	expressed in non-metastatic cells 1
rc_AI639141_at	rat mixed-tissue library rattus norvegicus cdna clone rx05003 3`, mrna sequence [rattus norvegicus]
rc_AI639534_g_at	rattus norvegicus transcribed sequence with moderate similarity to protein pir:s16150 (h.sapiens) s16150 properdin precursor - human
S46785_g_at	insulin-like growth factor binding protein complex acid-labile subunit [rats, liver, mrna, 2190 nt]
S70803_at	clone p10.15 product [rats, osteosarcoma ros17/2.8, mrna, 737 nt]
S85184_at	cyclic protein-2=cathepsin l proenzyme [rats, sertoli cells, mrna, 1790 nt]
U05784_s_at	microtubule-associated proteins 1a/1b light chain 3
U17260_s_at	n-acetyltransferase 1 (arylamine n-acetyltransferase)
U39208_at	cytochrome p450 4f6
U55938_at	sialyltransferase 8 c
U75397UTR#1_s_at	u75397utr#1 rnkrox2 rattus norvegicus krox-24 mrna, 3` untranslated region, partial sequence
U75689_s_at	deoxyribonuclease i-like 3
U94340_at	adp-ribosyltransferase 1

X06769cds_g_at	x06769cds rncfosr rat c-fos mrna
X16038exon_s_at	x16038exon rnalph13 r.norvegicus gene encoding alkaline phosphatase, exon 13
X16273cds_at	serine (or cysteine) proteinase inhibitor, clade a, member 1
X56325mRNA_s_at	hemoglobin, alpha 1
X56729mRNA_g_at	calpastatin
X71127_at	complement component 1, q subcomponent, beta polypeptide
X96437mRNA_g_at	x96437mrna rnprg1 r.norvegicus prg1 gene
